# Lysosomal agents inhibit store-operated Ca^2+^ entry

**DOI:** 10.1242/jcs.248658

**Published:** 2021-01-27

**Authors:** Anthony J. Morgan, Antony Galione

**Affiliations:** Department of Pharmacology, University of Oxford, Mansfield Road, Oxford OX1 3QT, UK

**Keywords:** Ca^2+^, GPN, LMP, Orai1, Stim1, Lysosome

## Abstract

Pharmacological manipulation of lysosome membrane integrity or ionic movements is a key strategy for probing lysosomal involvement in cellular processes. However, we have found an unexpected inhibition of store-operated Ca^2+^ entry (SOCE) by these agents. Dipeptides [glycyl-L-phenylalanine 2-naphthylamide (GPN) and L-leucyl-L-leucine methyl ester] that are inducers of lysosomal membrane permeabilization (LMP) uncoupled endoplasmic reticulum Ca^2+^-store depletion from SOCE by interfering with Stim1 oligomerization and/or Stim1 activation of Orai. Similarly, the K^+^/H^+^ ionophore, nigericin, that rapidly elevates lysosomal pH, also inhibited SOCE in a Stim1-dependent manner. In contrast, other strategies for manipulating lysosomes (bafilomycin A1, lysosomal re-positioning) had no effect upon SOCE. Finally, the effects of GPN on SOCE and Stim1 was reversed by a dynamin inhibitor, dynasore. Our data show that lysosomal agents not only release Ca^2+^ from stores but also uncouple this release from the normal recruitment of Ca^2+^ influx.

## Abstract (precis)

**Summary:** Lysosomal agents uncouple ER Ca^2+^-release from store-operated Ca^2+^ entry, predominantly by inhibiting Stim1 oligomerization and its activation of Orai.

## INTRODUCTION

Membrane-permeant lysosomotropic agents have long been used to disrupt lysosomal function and include two distinct classes: peptides or cationic amphiphilic drugs. Peptides such as glycyl-L-phenylalanine 2-naphthylamide (GPN) and L-leucyl-L-leucine methyl ester (LLOMe) are thought to be cleaved by lysosomal proteases and result in lysosomal osmotic rupture and lysosomal Ca^2+^ release (Morgan et al., 2020). Lysosomotropic agents can evoke a compromise of the lysosomal membrane integrity, referred to as lysosomal membrane permeabilization (LMP) (Villamil Giraldo et al., 2014; Wang et al., 2018). LMP is increasingly implicated in various cell death pathways in response to diverse stress stimuli, be they pathophysiological or pharmacological (Villamil Giraldo et al., 2014; Wang et al., 2018).

Historically, lysosomotropic agents were considered a useful tool for selectively evoking Ca^2+^ release from lysosomes (Morgan et al., 2020), but the mechanism and selectivity for lysosomes has been challenged recently (Atakpa et al., 2019). Regardless of the precise mechanism, it is agreed that lysosomotropic agents also evoke Ca^2+^ release from the endoplasmic reticulum (ER) Ca^2+^ stores (Morgan et al., 2020), it is just unclear whether this is secondary to an action on lysosomes or not (Atakpa et al., 2019); either lysosomal Ca^2+^ release can act as a ‘trigger’ to recruit the ER Ca^2+^ store ‘amplifier’ via Ca^2+^-induced Ca^2+^ release (CICR) (Kilpatrick et al., 2013; Morgan et al., 2020), or else the lysosomotropic agents could act independently of lysosomes and trigger ER Ca^2+^-release directly by undefined mechanisms (Atakpa et al., 2019; Cheng et al., 2019).

We have extended these studies to investigate how these agents may interact with the ER Ca^2+^ store by directly monitoring the ER luminal [Ca^2+^]. This unexpectedly revealed that lysosomal agents substantially emptied the ER but, surprisingly, without recruiting store-operated Ca^2+^ entry (SOCE). We demonstrate that lysosomal agents uncouple ER Ca^2+^-store depletion from SOCE by inhibiting the influx pathway in multiple ways.

## RESULTS

In Cos-7 cells, we simultaneously recorded Ca^2+^ changes in the cytosolic and ER luminal compartments using genetically encoded reporters, GCaMP6s (K_d_ 148 nM) and R-CEPIA1er (K_d_ 565 µM). Under resting conditions, the ER is Ca^2+^-replete, but R-CEPIA1er was not saturated because the R-CEPIA1er signal could be further increased by overloading the cell with Ca^2+^ using ionomycin/10 mM extracellular Ca^2+^ to determine the F_max_ (the resting signal was 75±1% of the F_max_, *n*=162 cells). This confirms that the response range of the reporter is appropriate for monitoring even initial changes of the ER Ca^2+^ concentration ([Ca^2+^]_ER_).

**Fig. 1.**
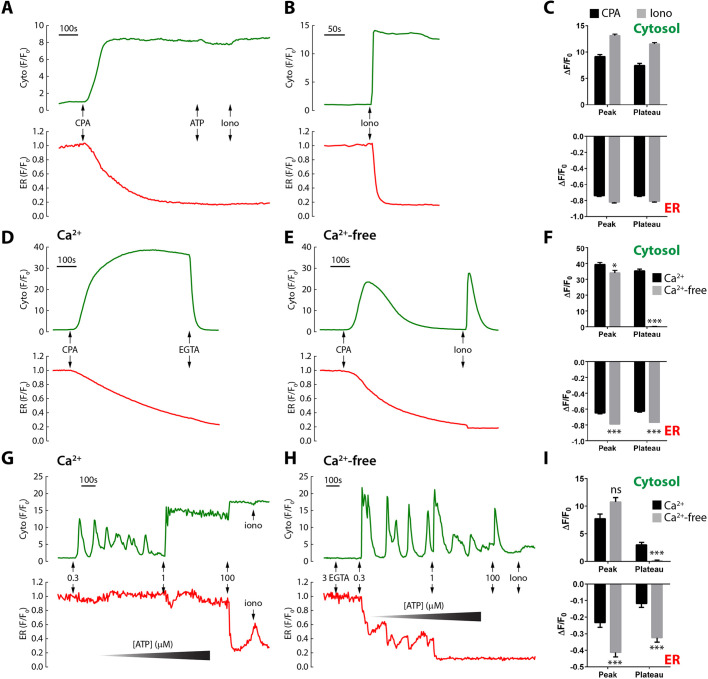
**Effect of ER Ca^2+^-mobilizing agents on cytosolic and ER Ca^2+^ levels.** Cells were co-transfected with GCaMP6s and R-CEPIA1er to simultaneously monitor cytosolic and ER luminal Ca^2+^, respectively. (A) Cells were stimulated as indicated with 50 µM CPA, 100 µM ATP and 1 µM ionomycin (iono). (B) Stimulation with 1 µM ionomycin. (C) Data are normalized to the initial fluorescence (F_0_) and responses quantified as the change (Δ) of the peak fluorescence, defined as the maximum deviation in each compartment, whereas the plateau is the average over its final 10-s period. Data are pooled from 174 cells (CPA) or 142 cells (ionomycin), and all responses are significantly different from basal levels (*N*=5-6, paired *t*-test, *P*<0.001). CPA responses in Ca^2+^-replete (D) or Ca^2+^-free medium (E); in each case, extracellular Ca^2+^ was chelated with 3 mM EGTA. Cumulative additions of ATP in Ca^2+^-containing (G) or Ca^2+^-free medium (H) produced by prior addition of 3 mM EGTA. (F,I) Peak responses to CPA or 0.3 µM ATP are quantified as the maximum response in each compartment, whereas the plateau is the average over its final 10-s period. Data are mean±s.e.m. of 282 (Ca^2+^) or 233 (Ca^2+^-free) cells, *N*=4 (CPA); 58 (Ca^2+^) or 83 (Ca^2+^-free) cells, *N*=3 (ATP). To compare responses in Ca^2+^ and Ca^2+^-free medium, *P* values were calculated using a non-parametric ANOVA (Dunn's). *P<0.05; ***P<0.001; ns, not significant.

To verify that R-CEPIA1er was performing as expected, we first applied stimuli known to deplete the ER directly, including the sarco-endoplasmic reticulum Ca^2+^-ATPase (SERCA) inhibitor, cyclopiazonic acid (CPA), ionomycin and the IP_3_-linked purinergic receptor agonist, ATP. Unsurprisingly, R-CEPIA1er showed antiparallel changes in [Ca^2+^]_ER_ compared with the cytosolic Ca^2+^ concentration ([Ca^2+^]_cyt_) as simultaneously monitored with GCaMP6s: CPA and ionomycin induced a complete emptying of the ER and an accompanying high cytosolic Ca^2+^ plateau (Fig. 1A-C). As expected, this plateau phase was due to SOCE because addition of extracellular EGTA either after (Fig. 1D,F) or before CPA (Fig. 1E,F) eliminated the cytosolic plateau phase. In contrast, ATP evoked submaximal responses and cytosolic Ca^2+^ oscillations (Fig. 1G). However, the impact on the ER levels depended on whether Ca^2+^ influx contributed or not: in Ca^2+^-containing medium, the fall in [Ca^2+^]_ER_ was small during these submaximal responses (Fig. 1G,I). In contrast, when Ca^2+^ influx was eliminated by extracellular EGTA, the cytosolic Ca^2+^ responses were broadly similar, but the fall in [Ca^2+^]_ER_ was significantly enhanced, and particularly marked for each successive cytosolic Ca^2+^ oscillation (Fig. 1H,I), i.e. Ca^2+^ influx helps to maintain the [Ca^2+^]_ER_ during ATP-induced spiking. Confident of the reporters, we first compared different lysosomal stimuli against their ability to recruit the ER.

### Lysosomal agents

**Fig. 2.**
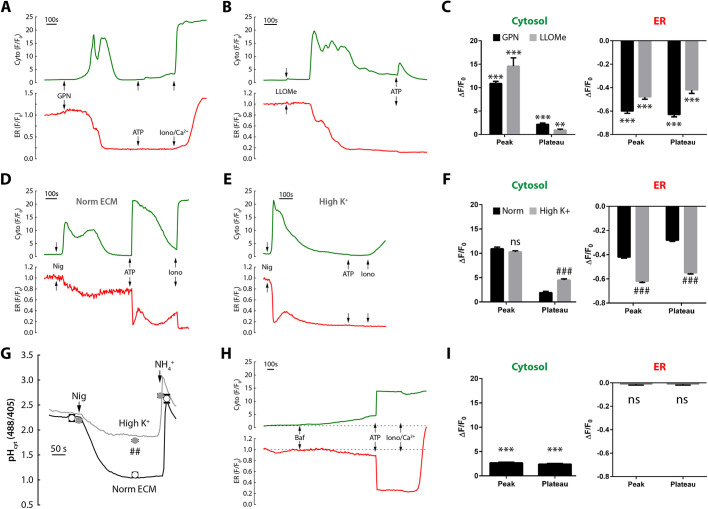
**Effect of lysosomal Ca^2+^-mobilizing agents on cytosolic and ER Ca^2+^ levels.** (A-F,H,I) Cells co-transfected with GCaMP6s and R-CEPIA1er were stimulated with 200 µM GPN (A), 5-10 mM LLOMe (B), 20 µM nigericin (D,E) or 1 µM bafilomycin A1 (H). Responses were normalized to the initial fluorescence (F_0_) and plotted as mean±s.e.m. for *n*=203 cells, *N*=8 (GPN); *n*=120, *N*=6 (LLOMe); *n*=242-471, *N*=6-10 (nigericin); *n*=121, *N*=6 (bafilomycin A1). The effect of high-K^+^ medium was investigated upon 20 μM nigericin responses to Ca^2+^ (D,E) or to cytosolic pH measured with BCECF (G, *n*=28-45, *N*=3). NH_4_Cl was added at 10 mM (G); the mean pH ratios (±s.e.m.) are superimposed in normal ECM (○) or in high-K^+^ ECM (●). **P<0.01, ***P<0.001 versus basal; ##*P*<0.01, ###*P*<0.001 versus corresponding normal medium. ns, not significant.

**Fig. 3.**
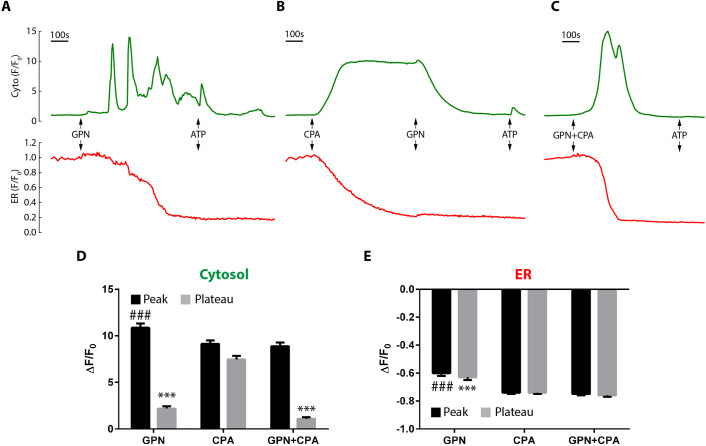
**Additivity of the effect of GPN and CPA on cytosolic and ER Ca^2+^ levels.** (A-C) Cells co-transfected with GCaMP6s and R-CEPIA1er were stimulated as indicated with 200 µM GPN, 50 µM CPA and 100 µM ATP. (A) GPN added alone; (B) CPA added before GPN; (C) GPN and CPA added simultaneously. (D,E) Data are normalized to the initial fluorescence (F_0_) and responses of the first addition quantified as the change (Δ) of the peak fluorescence, defined as the maximum deviation in each compartment, whereas the plateau is the average over its final 10-s period. The *x*-axis labels correspond to the responses to GPN (A), CPA (B) and GPN+CPA (C); *n*=132-203 cells, *N*=4-8. *P*<0.001 (###, compared to CPA peaks; ***, compared to CPA plateau), ANOVA (Tukey's test). Data are mean±s.e.m.

We first examined the LMP dipeptides, GPN and LLOMe. After a noticeable lag, GPN and LLOMe evoked large, transient increases in [Ca^2+^]_cyt_ that returned close to basal levels (Fig. 2A-C). Individual cells differed in their response pattern, but [Ca^2+^]_cyt_ oscillations were often observed (compare Fig. 2A and Fig. 3A). In terms of amplitude, the [Ca^2+^]_cyt_ spikes elicited by the LMP agents were similar to those of ATP. Why LMP-agent [Ca^2+^]_cyt_ responses run down has hitherto been unclear.

Indirect evidence has previously suggested that these agents also deplete the ER (Atakpa et al., 2019; Kilpatrick et al., 2013; Ronco et al., 2015), but we now employ real-time recordings of R-CEPIA1er to directly confirm ER mobilization by GPN/LLOMe: both peptides evoked a mobilization of the ER Ca^2+^ store as judged by the stepwise fall in R-CEPIA1er (and confirmed by the lack of further effect of maximal ATP; Fig. 2A-C). Note that GPN evoked an initial small increase in the R-CEPIA1er signal, but this was unlikely to be due a real Ca^2+^ increase because it was also observed in the presence of CPA, which inhibits Ca^2+^ uptake into the ER (Fig. 3B), and may relate to a transient cellular pH change evoked by GPN (Atakpa et al., 2019). What was unexpected was that GPN and LLOMe both induced a sustained and substantial depletion of the ER (Fig. 2A-C); considering that these experiments were performed in Ca^2+^-containing medium, it was surprising that this was not accompanied by a SOCE plateau and the cytosolic Ca^2+^ responses simply returned to near basal levels. This suggested that SOCE was uncoupled from the manifest depletion of the ER.

To test whether this uncoupling of SOCE was specific to lysosomotropic agents that can induce LMP, we compared the effect of nigericin, an electroneutral ionophore that exchanges K^+^ and H^+^, which is used to target lysosomal Ca^2+^ stores by increasing their luminal pH (pH_L_) (Fasolato et al., 1991; Katsnelson et al., 2016). In spite of a different mechanism of action from GPN/LLOMe, nigericin also stimulated anti-parallel changes in the [Ca^2+^]_cyt_ and [Ca^2+^]_ER_ (Fig. 2D,F), and sometimes oscillatory (data not shown). Moreover, the later cytosolic Ca^2+^-entry phase was small and similar in amplitude to that evoked by GPN, even though the ER was manifestly depleted. Superficially, this was consistent with GPN and nigericin having similar effects upon SOCE.

**Fig. 4.**
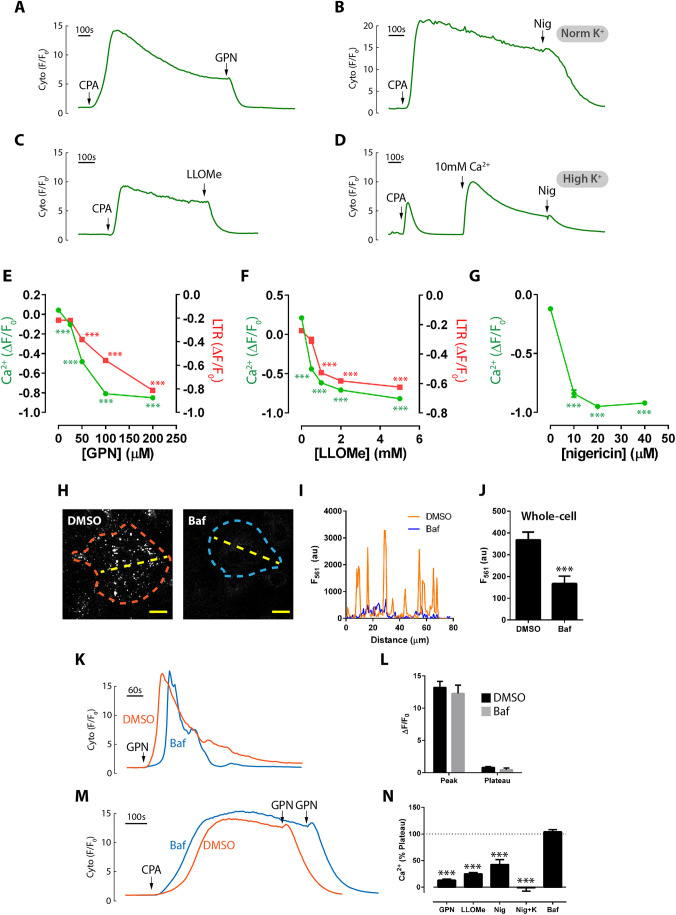
**Effect of lysosomal agents and chronic bafilomycin A1 on SOCE.** (A-D) SOCE monitored with GCaMP6s was stimulated with 50 µM CPA and then different agents were added to the plateau phase [200 µM GPN (A), 20 µM nigericin (B,D) and 5 mM LLOMe (C)]. In D, cells were incubated in ECM with high K^+^. (E-G) Concentration dependence of agents action upon SOCE (Ca^2+^) or lysosomes (Lysotracker Red labelling, LTR) amplitudes monitored simultaneously (*n*=82-303, *N*=3-4; ***P<0.001 versus vehicle control, Dunn's multiple comparison). (H) Cells were treated with 0.1% DMSO or 1 µM bafilomycin A1 for 60 min and then loaded with 300 nM Lysotracker Red for 5 min. Lysotracker Red labelling was collected with the same acquisition settings for DMSO/bafilomycin A1 (Baf; orange/blue-dotted lines show single-cell boundaries). Scale bars: 20 µm. (I) Profile plot of fluorescence across the yellow-dashed lines in the images in H. (J) Whole-cell Lysotracker Red fluorescence of cells in H (*n*=28-44 cells, *N*=3,***P<0.001 versus DMSO). (K) GCaMP6s-expressing cells were pretreated as in H and then stimulated with 200 µM GPN. (L) Quantification of GPN responses in K (*n*=21-34 cells, *N*=3). (M) Cells were pre-incubated with or without bafilomycin A1 as in H and 50 µM CPA and 200 µM GPN applied as indicated. (N) Data normalized to the CPA plateau in untreated cells (*n*=39-188 cells, *N*=3-9). Data are mean±s.e.m. ***P<0.001 (paired Student's *t*-test versus pre-addition plateau).

However, other considerations must be taken into account. Most obviously, as an ionophore, nigericin is not selective for lysosomes but also acts at other membranes, including the plasma membrane. With nigericin in the plasma membrane, the K^+^ gradient drives H^+^ uptake and an acidification of the cytosol, as we confirmed using the cytosolic pH probe, 2′,7′-bis-(2-carboxyethyl)-5(and-6) carboxyfluorescein (BCECF) (Fig. 2G), which was reversed by addition of NH_4_Cl (note that the time-to-peak of Ca^2+^ release is faster than of acidification). For comparison, this contrasts with the smaller transient alkalinization induced by GPN (Atakpa et al., 2019). We then reasoned that if we reduced the K^+^ gradient across the plasma membrane, then this would decrease the nigericin-induced acidification of the cytosol. This proved correct as cells in a high-K^+^ medium (145 mM) showed a significant (∼70%) reduction in the nigericin-induced acidification BCECF signal (Fig. 2G), without the basal cytoplasmic pH being affected. Returning to measurements of cytosolic and ER Ca^2+^, nigericin in high-K^+^ medium evoked similar Ca^2+^ spiking to that observed in normal K^+^ (Fig. 2E,F); indeed, the excursions in both [Ca^2+^]_cyt_ and [Ca^2+^]_ER_ were actually enhanced by high-K^+^, implying that Ca^2+^ signals were merely dampened by acidification and not actually driven by it (the discrepancy between the rapid Ca^2+^ and slow pH kinetics also argued against causality). These results imply two things: (1) that these Ca^2+^ responses are mainly driven by nigericin acting on internal membranes; (2) the cytosolic Ca^2+^ spikes are predominantly due to release from internal stores (including the ER) because Ca^2+^ influx is inhibited in high K^+^ due to membrane depolarization (see Fig. 4D).

Finally, we applied acutely the lysosomal V-H^+^-ATPase inhibitor, bafilomycin A1 which stimulates lysosomal Ca^2+^ release in some cell types (Davis et al., 2012; Lloyd-Evans et al., 2008). In contrast to the other lysosomal agents, bafilomycin A1 failed to evoke robust Ca^2+^ signals, instead only promoting a slow and small increase in [Ca^2+^]_cyt_ over 20 min with little detectable mobilization of the ER (Fig. 2H,I). Because bafilomycin A1-induced Ca^2+^ signals would require both H^+^ leak and Ca^2+^ leak (Morgan et al., 2020), the data suggest either that leak rates are low in these cells or that lysosomes do not fill via a pH-dependent mechanism (Garrity et al., 2016). Either way, bafilomycin A1 is a poor tool for stimulating Ca^2+^ signals in this cell type. Taken together, all the lysosomal agents studied act upon organelles, mobilize the ER Ca^2+^-store (bafilomycin A1 excepted), but poorly stimulate Ca^2+^ entry.

### Agents and ER stores

We had demonstrated that the agents mobilized the ER but that SOCE did not accompany this depletion, so we examined this in more detail. First, we examined how GPN and the SERCA inhibitor, CPA, affected one another, either sequentially or simultaneously. Fig. 3A reaffirms that GPN alone elicits complex Ca^2+^ spiking that depletes the ER stepwise (indicated by R-CEPIA1er and the poor subsequent ATP response). In contrast, CPA evokes a slow release from stores that resulted in a sustained cytosolic Ca^2+^ response due to SOCE (Fig. 3B); however, when GPN was subsequently added to the SOCE phase, there was a prompt reduction of the cytosolic plateau, but without any effect upon the ER Ca^2+^ levels (Fig. 3B), mimicking the effect of adding extracellular EGTA (Fig. 1D); again, the empty ER failed to support a subsequent ATP response (Fig. 3B). This is the first evidence that GPN inhibits the SOCE phase independently of its effects upon ER Ca^2+^ emptying and, importantly, inhibits SOCE after it has already been activated, i.e. it reverses SOCE. Finally, we added CPA and GPN simultaneously, and, again, this evoked a transient cytosolic Ca^2+^ spike that fully depleted the ER without SOCE (Fig. 3C). These data show that GPN inhibits SOCE independently of effects upon the ER Ca^2+^-store filling and irrespective of whether there was a prior or concurrent activation of SOCE by CPA (Fig. 3D,E).

Similar results were observed with LLOMe and nigericin. When SOCE was established by CPA, addition of GPN, LLOMe or nigericin rapidly and profoundly inhibited the Ca^2+^ plateau (Fig. 4A-C,N) in a concentration-dependent manner (Fig. 4E-G; Fig. S1C,D). To ensure that the inhibition by nigericin was not simply due to cytosolic acidification, we repeated the protocol in high-K^+^ medium (see Fig. 2G). In high K^+^, the CPA response itself was rendered transient because the driving force for SOCE is reduced by plasma membrane depolarization (Fig. 4D). Without discernible SOCE, it was impossible to assess the effect of nigericin, so we added a further 10 mM extracellular Ca^2+^ to drive a detectable level of Ca^2+^ entry (Fig. 4D); to this new plateau, we added nigericin and continued to observe the inhibition of Ca^2+^ entry, even in high K^+^ (Fig. 4D,N). In summary, all three agents rapidly inhibit SOCE.

### Role of lysosomes

Thus far, we have described the inhibition of SOCE by these agents but have not resolved a mechanism or target(s). Until recently, GPN and LLOMe were thought to act by inducing LMP (Morgan et al., 2020), although this mechanism has been challenged (Atakpa et al., 2019). The first obvious question is: are these lysosomal agents acting via lysosomes? It is currently technically difficult to definitively answer this (Morgan et al., 2020), but we performed a panel of experiments to try to address this. First, we compared the concentration-response relationship between the effect on SOCE and on lysosomes (as judged by the fall in the fluorescence of Lysotracker Red, a lysosome-specific stain that is eliminated by compromised luminal pH or membrane integrity – Fig. S1A,B). Broadly speaking, the effect of GPN and LLOMe on lysosomes occurred over similar concentration ranges as the effect on SOCE, although whether it was the magnitude of inhibition (Fig. 4E,F) or its kinetics (Fig. S1C,D) that showed closer correspondence differed between GPN and LLOMe. Unfortunately, with nigericin, we could not systematically quantify the Lysotracker fluorescence changes: although nigericin reassuringly eliminates lysosomal labelling, the Lysotracker translocates and is retained by other endomembranes so that the overall mean fluorescence is not reduced (Fig. S1F-I). Overall, the potency of the effect of the agents on SOCE overlaps with the potency at lysosomes.

Probing lysosomes in another manner, we specifically inhibited the lysosomal H^+^ pump (V-ATPase) with bafilomycin A1, to increase the lysosomal pH_L_ and, possibly, to deplete the lysosomes of Ca^2+^ indirectly (albeit controversially) (Morgan et al., 2011; Yang et al., 2019). As Cos-7 cells barely responded to acute application of bafilomycin A1 in terms of Ca^2+^ release (Fig. 2H,I), we pre-incubated cells with bafilomycin A1 for 60 min, a condition that eliminated punctate Lysotracker Red staining (Fig. 4H,I), which indicates a collapse of the lysosomal pH gradient; note that the significant reduction in whole-cell Lysotracker fluorescence (Fig. 4J) is an underestimate of the effect owing to a partial retention by other endomembranes (though less marked than that observed with nigericin above). Conditions defined, GPN-evoked Ca^2+^ responses were unaffected by bafilomycin A1, neither the peak nor the plateau (Fig. 4K,L), and others have also found that GPN-evoked Ca^2+^ release is insensitive to bafilomycin A1 (Atakpa et al., 2019). In contrast to acute lysosomal agents, chronic bafilomycin A1 treatment did not affect the SOCE plateau evoked by CPA (*P*>0.2; Fig. 4M,N). Moreover, GPN could still inhibit the SOCE phase even in these cells pretreated with bafilomycin A1 (Fig. 4M; % inhibition by GPN: DMSO 81±4%, bafilomycin A1 101±1%, *P*<0.001 paired *t*-tests versus respective plateau phases). These data suggest that lysosomal pH_L_ does not play a major role in regulating SOCE: chronically increasing lysosomal pH_L_ does not per se inhibit SOCE, nor does it affect the ability of GPN to block SOCE (i.e. the GPN effect is pH_L_-independent).

**Fig. 5.**
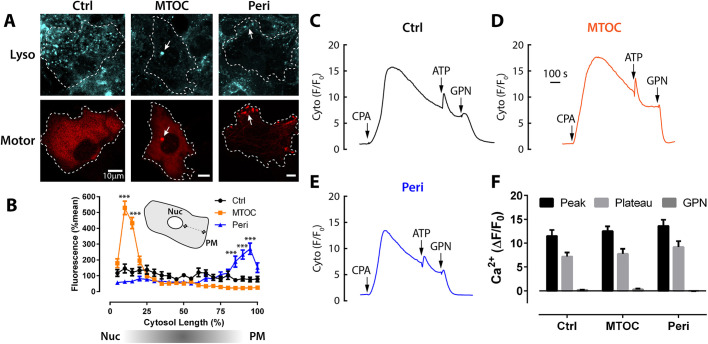
**Effect of lysosome repositioning on SOCE.** (A-F) Cells were transfected with a rapalog-induced dimerization system that crosslinks lysosomes to molecular motors to drive vesicle repositioning. Cells were triple transfected with GCaMP6s, LAMP1-ECFP-FRB* plus either tdTomato-BicD2-FKBP12 or KIF5C-tdTomato-FKBP12 to drive lysosomes to the MTOC or periphery, respectively. Dimerization and movement was initiated by incubating cells with 250 nM AP21967 (rapalog) for 90-120 min. Ethanol (0.05%) was used as control. Cells were counterstained with 300 nM Lysotracker Deep Red for 5 min. Single cells were selected with demonstrable repositioning. (A) FKBP12-motor-binding proteins (Red) and Lysotracker Deep Red (Lyso, Cyan) images showing control (Ctrl, ethanol treated, A) or rapalog-treated cells (MTOC, Peri). White-dashed lines indicate single-cell boundaries, and arrows highlight equivalent regions of aggregated lysosomes in each channel. (B) Distribution of the lysosomal fluorescence along a profile intensity plot between the nucleus and plasma membrane. For clarity, only peak fluorescence significances versus control are indicated (*n*=11-23, *N*=4-9; **P<0.01, ***P<0.001, respectively). (C-E) Ca^2+^ recordings on a common time scale (shown in D), whereby SOCE was promoted by the addition of 50 µM CPA (ER depletion was confirmed with 100 µM ATP) and 200 µM GPN. (F) Bar chart showing Ca^2+^ levels at peak and plateau phases, before and after addition of GPN, in control cells or in cells with lysosomes moved to the MTOC or periphery. Data are mean±s.e.m. (*n*=12-16 cells; *N*=5).

Finally, the proximity of lysosomes to the plasma membrane can alter their ability to modulate SOCE (Sbano et al., 2017), so we likewise investigated whether lysosomal repositioning and clustering altered SOCE and/or its sensitivity to GPN. To do this, we acutely tethered lysosomes to dynein or kinesin motor proteins using the FKBP12/FRB* system (Bentley et al., 2015) dimerized by a rapalog (AP21967, which is not an mTOR inhibitor). Lysosomes were either moved centripetally to the microtubule organizing centre (MTOC; via dynein) or centrifugally to the cell periphery (via kinesin) (Fig. 5A,B), and vesicle translocation could be visualized in three ways: by Lysotracker, the LAMP1 bait itself and the vesicle-associated motor-binding protein (Fig. 5A, Fig. S2). Monitoring Ca^2+^, we found that the CPA-evoked SOCE phase was itself unaffected by lysosomal repositioning (Fig. 5C-F) and, moreover, that the GPN inhibition of SOCE also persisted when lysosomes were moved out of position (Fig. 5C-F). Taken together, this set of data suggests that neither bafilomycin A1 nor lysosomal positioning affect SOCE per se or the ability of GPN to inhibit SOCE.

### Lysosomal agents inhibit the Stim1 pathway

We probed further which aspect(s) of the SOCE pathway was inhibited by the lysosomal agents. In the simplest model, the depletion of the ER Ca^2+^ store is sensed by the ER luminal Ca^2+^ sensor, Stim1, which then oligomerizes and unfolds so that its newly exposed STIM-Orai activating region (SOAR) contacts and activates the plasma membrane Orai (herein not referring to a specific isoform unless otherwise mentioned) channel family (Soboloff et al., 2012). We therefore investigated whether the lysosomal agents interfered with elements along this pathway.

**Fig. 6.**
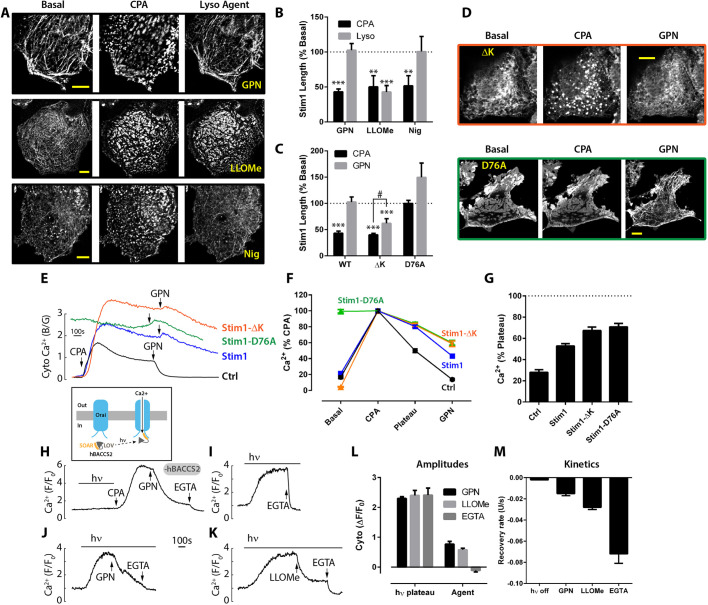
**Lysosomal agents and Stim1 signalling.** (A-D) Cells transfected with EYFP-tagged Stim1 (or mutants) were treated with 50 µM CPA for 15-20 min to initiate SOCE, and then lysosomal agents were added acutely (200 µM GPN, 5 mM LLOMe and 20 µM nigericin). Binary threshold masking determined the length of the Stim1 structures (length before GPN=13.7±3.0 µm). (A) Wild-type EYFP-Stim1 before and 5 min after agents were added (GPN, *n*=21, *N*=16; LLOMe, *n*=7, *N*=5; and nigericin, *n*=5, *N*=3). (B) The cell-average length of Stim1 structures was normalized to the basal value. (C) Quantification of the effect of 200 µM GPN on the length of structures formed by Stim1 variants, wild type (WT, *n*=21, *N*=16), Stim1-ΔK (*n*=9, *N*=3) and Stim1-D76A (*n*=13 cells, *N*=3). (D) Morphology of Stim1 mutants (Stim1-ΔK and Stim1-D76A) treated with 50 µM CPA then 200 µM GPN. (E-G) Cells were co-transfected with (or without) EYFP-tagged Stim1 (or mutants) and the ratiometric Ca^2+^ reporter GEM-GECO1. SOCE was initiated with 50 µM CPA, and then 200 µM GPN was acutely applied. (E) Representative single-cell Ca^2+^ traces. (F) Collated data expressed as percentage of the CPA peak ratio. (G) Plateau phase as a percentage of the pre-GPN value. Data are mean±s.e.m. of 67-86 cells (*N*=3-9). (H-M) Effect of lysosomal agents on SOCE evoked optogenetically by hBACCS2 (see inset cartoon) activated with 488 nm light (indicated by the bar, hν), as measured with the red Ca^2+^ reporter JRGECO1a. (H) Cells expressing the EGFP transfection marker alone. (I-K) Cells co-expressing EGFP plus hBACCS2. Ca^2+^ influx was inhibited by 3 mM EGTA, 200 µM GPN or 5 mM LLOMe. (L) Ca^2+^ amplitudes before and after agent addition. (M) Rate of fall in JRGECO1a signal upon removal of light (hν off) or addition of agents. Data are mean±s.e.m. of 22-101 cells (*N*=3-4). **P<0.01, ***P<0.001 (ANOVA, Tukey-Kramer post-test). Scale bars: 10 μm.

First, we monitored the oligomerization of Stim1 to see whether it was altered, using imaging with confocal slices collected at the plasma membrane-substratum interface. In resting cells expressing EYFP-Stim1, we observed its characteristic ER/microtubular morphology comprised of elongated structures. Depletion of the ER stores with CPA provoked the expected EYFP-Stim1 puncta formation, indicative of oligomerization (Fig. 6A) and quantified as a decrease in Stim1 length (Fig. 6B), area and perimeter (Fig. S3A,B), or an increase in shape circularity (Fig. S3C). Remarkably, GPN addition rapidly reversed this aggregation and restored the resting ER/microtubular morphology (Fig. 6A,B; Fig. S3A-C). Similarly, nigericin also dispersed Stim1 puncta (Fig. 6A,B; Fig. S3A-C). In contrast to the effects of nigericin and GPN on Stim1 oligomerization, LLOMe failed to disperse Stim1 puncta (Fig. 6A, middle panels; Fig. 6B). Therefore, although GPN/nigericin may inhibit SOCE in part by reversing Stim1 oligomerization, LLOMe must be acting elsewhere.

Second, we investigated the susceptibility of Stim1 variants (Liou et al., 2005), either with a deletion of the polybasic domain that normally binds to plasma membrane phospholipids (ΔK) or with a mutation of the luminal Ca^2+^-sensing EF-hand (D76A). As with the wild-type Stim1, GPN reversed the CPA-induced aggregation of the EYFP-Stim1ΔK variant (Fig. 6C,D), although the effect was not quite so marked as with the wild-type Stim1. Finally, the Ca^2+^-sensor mutant, D76A, is already constitutively aggregated because the mutation mimics Ca^2+^-emptying of the ER (Liou et al., 2005). Interestingly, GPN also drove this EF-mutant Stim1 into an ER/microtubular morphology and promoted disassembly of the oligomers (Fig. 6C,D). Because the D76A aggregates were so large from the outset (regardless of CPA), the reversal by GPN was less ably detected using this type of analysis (Fig. 6C), and although there was a tendency to observe a GPN-induced increase in length of Stim1, it did not reach significance (*P*=0.10), even though the morphological changes were clear (Fig. 6D, lower panels). Regardless of the Stim1 variant, GPN dispersed aggregated Stim1.

Further evidence implied that GPN acted via a Stim1-dependent pathway. Monitoring Ca^2+^ ratiometrically with GEM-GECO1, we tested the effect of expressing EYFP-Stim1 variants upon CPA and GPN Ca^2+^ signals (Fig. 6). In cells with Stim1 at endogenous levels, CPA evoked a SOCE phase that was robustly inhibited by GPN (Fig. 6E). However, in cells with high levels of EYFP-Stim1 expression, not only was the Ca^2+^-influx phase enhanced as expected, but GPN was a weaker inhibitor of the SOCE phase (Fig. 6E-G). That is, overexpressing Stim1 partially overcame the inhibition. The ΔK mutant similarly prevented GPN from inhibiting the SOCE phase (Fig. 6E-G). When using the constitutively active D76A mutant, the resting [Ca^2+^] was elevated, consistent with constitutive Orai activation; again, GPN was a weaker SOCE blocker (Fig. 6A-C). In summary, overexpression of any Stim1 variant functionally protected the cell from the inhibition by GPN. One interpretation is that Stim1 activity is affected by GPN and that its overexpression compensates for this.

We then addressed whether the agents could inhibit downstream of Stim1 aggregation/unfolding, i.e. interfere with Stim1 interaction with Orai. To this end, we stimulated Orai channels optogenetically with a cytosolic SOAR fragment of Stim1 that is caged in the dark with the LOV2-Jα domain [hBACCS2 (Ishii et al., 2015); see cartoon Fig. 6H]. In cells expressing just the transfection marker EGFP, the uncaging blue light had no effect upon cytosolic Ca^2+^ measured with a red GECI (Fig. 6H). When hBACCS2 was co-expressed, blue light evoked a slow increase in Ca^2+^ that reached a plateau (Fig. 6I-K) and returned back to basal levels slowly when blue light was removed (Fig. 6M); the response was driven by Ca^2+^ influx because chelation of extracellular Ca^2+^ with EGTA rapidly eliminated it (Fig. 6H-M), indicating that Orai had been activated by uncaging SOAR. Turning to the effect of lysosomal agents, we found that both GPN and LLOMe profoundly inhibited hBACCS2-dependent responses (Fig. 6J-L); GPN and LLOMe both promoted a decrease in Ca^2+^ that was faster than that seen upon the removal of activating light, but not as fast as the effect of EGTA (Fig. 6M). This is consistent with both lysosomal agents inhibiting SOCE downstream of Stim1 activation, e.g. by interrupting SOAR-Orai interactions.

### Membrane events

**Fig. 7.**
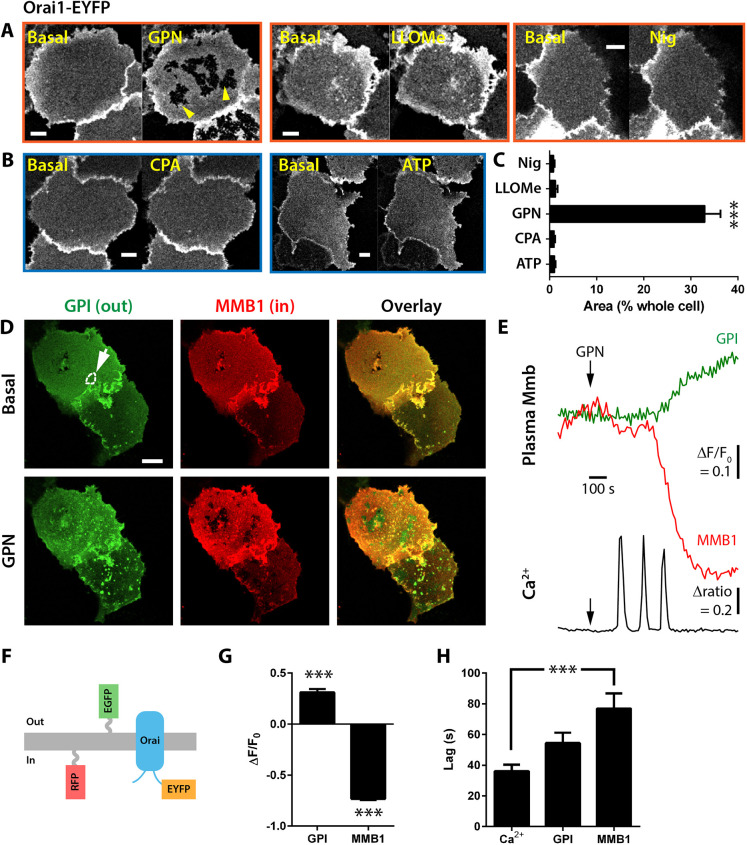
**GPN leads to plasma-membrane protein redistribution.** (A) Orai1-EYFP labelling is disrupted by 200 µM GPN (5 min), with formation of dark patches (highlighted by yellow arrowheads). In contrast, neither the lysosomal agents (2 mM LLOMe and 20 µM nigericin) nor ER agonists (B; 50 µM CPA and 100 µM ATP) evoked patch formation. (C) Patch size expressed as a percentage of the whole-cell area (*n*=7-31, *N*=7-14; paired *t*-test versus pre-stimulation). Raw data for the GPN-treated cells were as follows: whole-cell, 1967±203 µm^2^; and patches 620±73 µm^2^ (*n*=31, *N*=14). (D-H) Simultaneous recording of outer (GPI-EGFP) and inner (TagRFP-T-Membrane1) plasma membrane morphology and [Ca^2+^]_cyt_ with GEM-GECO1. (D) Images showing plasma membrane labelling before and after 200 µM GPN treatment. (E) Time-course of the fluorescence of plasma membrane labels normalized to the pre-GPN intensity (F/F_0_) corresponding to the dotted region of interest drawn on the basal GPI channel (highlighted by the arrow in D). Lower GEM-GECO1 ratio Ca^2+^ signal for the same ROI. (F) Cartoon indicating topology of plasma-membrane labels. (G) Amplitude of fluorescence changes in the outer (GPI) and inner (MMB1) at ‘patch sites’. (H) Lag time between addition of GPN and the first discernible fluorescence change (*n*=16, *N*=11). Data are mean±s.e.m. ***P<0.001 (paired *t*-test versus basal, G; paired ANOVA, Tukey–Kramer, H). Scale bars: 20 µm.

In addition to implicating Stim1, we tested whether lysosomal agents also affected Orai. For example, could these lysosomal agents reduce Orai interactions with Stim1 by promoting a redistribution of Orai away from Stim1 puncta? First, we imaged Orai1-EYFP. To our surprise, the addition of GPN led to a substantial redistribution of Orai1 in the plane of the membrane such that dark patches appeared, presumably due to occlusion of Orai1-EYFP from these areas (Fig. 7A,C). GPN was apparently unique in this because treatment with the other lysosomal agents LLOMe or nigericin did not induce areas of occlusion (Fig. 7A,C), and neither did the ER agents, CPA or the purinoceptor agonist ATP (Fig. 7B,C). Further work showed that these patches were not specific to Orai1 but represented a general perturbation of the plasma membrane: another protein, mTagRFP-Membrane-1, is tethered to the plasma membrane inner-leaflet by palmitoylation (Fig. 7F), and this exhibited a similar rapid redistribution and dark-patch formation in response to GPN (Fig. 7D,E,G). Such a GPN-induced occlusion was specific for the inner leaflet of the plasma membrane because simultaneous labelling of the outer leaflet with a glycosylphosphatidylinositol (GPI)-anchored GFP (Fig. 7F) did not form dark patches but rather tended to form bright puncta (Fig. 7D,E,G). As for the relative kinetics, simultaneously monitoring [Ca^2+^]_cyt_ with GEM-GECO1 revealed that Ca^2+^ release from stores preceded both the inner and outer leaflet rearrangements (Fig. 7H). We conclude that GPN (but not other Ca^2+^-releasing agents) evokes a redistribution of plasma membrane proteins, including Orai1, via changes to the inner leaflet, which is likely to reduce the interaction interface area with Stim1.

**Fig. 8.**
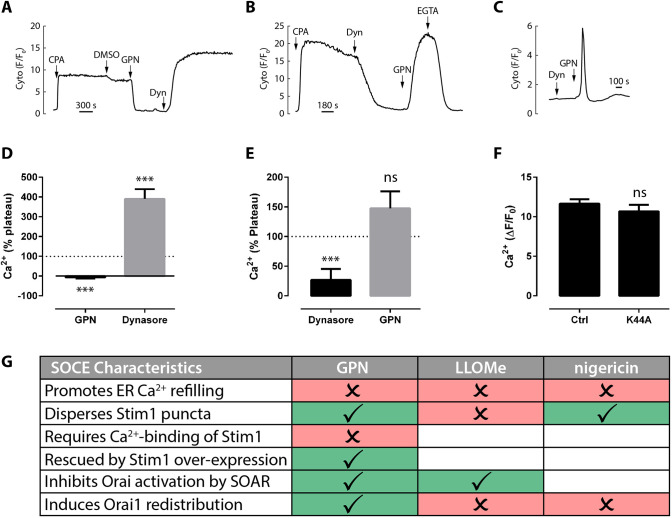
**GPN-induced inhibition of SOCE is reversed by dynasore.** SOCE was initiated in cells transfected with GCaMP6s by applying 50 µM CPA. Subsequently, 0.1% DMSO, 200 µM GPN, 80 µM dynasore or 3 mM EGTA were applied as indicated. Data are quantified as a percentage of the preceding CPA-induced SOCE plateau (D,E). (A,D) GPN added before dynasore. (B,E) Dynasore added before GPN. (C) Dynasore and GPN added to naïve cells (without CPA). Data are mean±s.e.m. of 73-139 cells, *N*=3-6. (F) The CPA SOCE plateau-phase in cells transfected with or without the dominant-negative mutant of dynamin-2K44A (*n*=39-40 cells, *N*=3). ***P<0.001 (paired ANOVA versus plateau); ns, not significant (*P*>0.1). (G) Table summarizing the effect of the lysosomal agents on different aspects of SOCE. Red ⨯, no requirement or effect; green ✓, requirement or effect. Blank cells, not determined.

Because plasma membrane levels of Orai1 can be regulated by dynamin-mediated endocytosis (Yu et al., 2010), we wondered whether lysosomal agents might also reduce Orai by this pathway. If GPN was promoting Orai endocytosis, blocking dynamin should reverse the GPN effect. First we applied the dynamin inhibitor, dynasore (Basagiannis et al., 2017; Preta et al., 2015). Dynasore alone had no effect upon resting Ca^2+^ levels nor prevented GPN-induced Ca^2+^ release (Fig. 8C). However, addition of dynasore after GPN promptly reversed the GPN block of SOCE and a robust Ca^2+^ entry was restored (Fig. 8A,D). Although this was consistent with our endocytosis hypothesis, the situation appeared to be more complex. When we reversed the order of addition of the two agents, dynasore by itself now inhibited the SOCE phase (Fig. 8B,E); conversely, subsequent application of GPN promptly reversed this inhibition (Fig. 8B,E). That is, both GPN and dynasore alone could block SOCE, but they were mutually antagonistic and subsequently rescued the other. Dynasore is well known to act at sites other than dynamin (Basagiannis et al., 2017; Preta et al., 2015) so we also tested the effect of genetic inhibition of dynamin on SOCE by overexpressing a dominant-negative mutant of dynamin-2 (K44A). In contrast to the dynasore inhibition of SOCE, the K44A mutant had no effect upon the SOCE plateau evoked by CPA (Fig. 8F). Together, the data indicate that GPN can be functionally antagonised by dynasore, but this is likely to be at an unknown off-site target, and unlikely to be via an effect on dynamin-dependent endocytosis. The effects of the lysosomal agents are summarized in Fig. 8G.

## DISCUSSION

Cytosolic signals are the complex summation of multiple Ca^2+^-stores, channels and removal processes, including bidirectional interactions between lysosomes and ER Ca^2+^ stores. With similar luminal [Ca^2+^], lysosomes and ER contribute to cytosolic signals in proportion to their total volume; accordingly, the small lysosomal volume equates to small cytosolic signals (often undetectable in global Ca^2+^ recordings; Davis et al., 2020), whereas it is the large ER (and accompanying SOCE) that generates the majority of the detectable cytosolic Ca^2+^. However, the stimulation of lysosomal Ca^2+^ release demonstrably evokes substantial Ca^2+^ signals and this paradox has led to the ‘trigger hypothesis’, whereby the small (‘invisible’) lysosomal Ca^2+^ release is a ‘trigger’ that secondarily recruits the ‘amplifier’, the major ER Ca^2+^ store (Davis et al., 2020; Kilpatrick et al., 2013; Morgan et al., 2020). This hypothesis has, in part, been supported by the use of lysosomal agents, such as GPN, LLOMe and nigericin that evoke disproportionally large Ca^2+^ responses indicative of ER Ca^2+^ release.

This current study stemmed from the initial observation that agents used to mobilize lysosomal Ca^2+^ evoke substantial Ca^2+^ spiking, but these run down, even in Ca^2+^-containing medium. A reasonable and obvious interpretation might have been that the lysosomal agents have irreversibly depleted the lysosomal Ca^2+^ stores – without the trigger, the amplifier switches off and therefore Ca^2+^ returns to resting levels. However, by simultaneously monitoring the ER [Ca^2+^], we unexpectedly found that these agents substantially depleted the ER, and this was the main reason why the spiking stopped. Nigericin and GPN were previously shown to deplete the ER using a lumen-targeted aequorin (Ronco et al., 2015), although this was not able to reveal their acute real-time kinetics. What was puzzling was that there was no SOCE even though the ER stores were depleted. We therefore hypothesised that these agents were not only evoking ER Ca^2+^ release but, in parallel, were inhibiting SOCE.

### Inhibition of SOCE

Multiple lines of evidence suggest that lysosomal agents inhibit SOCE: (1) they deplete ER Ca^2+^ stores without a sustained elevation of cytosolic Ca^2+^; (2) the SOCE plateau phase induced by the SERCA inhibitor, CPA, is acutely inhibited by these agents; and (3) circumstantially, when we compare GPN- and ATP-induced Ca^2+^ oscillations, the pattern of ER Ca^2+^ oscillations with GPN more closely resembles that with ATP in Ca^2+^-free medium, i.e. ER Ca^2+^ does not recover well per spike, but proceeds as a stepwise depletion. This is consistent with SOCE normally contributing to the replenishment of ER Ca^2+^ stores during spiking, but this occurs poorly with lysosomal agents. Furthermore, we consider it unlikely that the repression of the SOCE phase by lysosomal agents is merely due to enhanced Ca^2+^ removal: the agents inhibit the SOCE phase promptly and persistently, and yet these agents do not repress their own Ca^2+^ release when applied alone.

### Loci of SOCE blockade

The inhibition of SOCE by lysosomal agents could occur at any of multiple points along the pathway and we consider the following in order: (1) ER Ca^2+^-store refilling; (2) Stim1 Ca^2+^-sensing; (3) Stim1 oligomerization; (4) Stim1 activation of Orai; and (5) Orai-pore block. First, we exclude ER Ca^2+^ store refilling because R-CEPIA1er recordings demonstrate that the ER remains depleted when GPN inhibits SOCE (Figs 2, 3); it is also unlikely that GPN is refilling a small subcompartment of the ER that is masked in global ER recordings (e.g. a compartment closely apposed to the plasma membrane that controls Orai) because refilling is globally blocked by SERCA inhibition.

Could GPN modulate the Ca^2+^-sensor of Stim1? For GPN to reverse SOCE by this mechanism, it would need to enhance (or mimic) Ca^2+^-binding to the Stim1 luminal Ca^2+^-binding sites, even when the Ca^2+^ store is depleted. Given that the R-CEPIA1er Ca^2+^ affinity is 565 µM (Suzuki et al., 2014) and the resting fluorescence is 75% of its F_max_, we estimate resting ER [Ca^2+^] in Cos-7 cells to be ∼1.5 mM and ‘empty’ stores to be <75 µM; for comparison, the Ca^2+^ affinity of Stim1 is ∼200 µM (Soboloff et al., 2012), and Stim1 puncta association/dissociation reported with *in situ* affinities of 350/530 µM, respectively (Suzuki et al., 2014). We consider it unlikely that GPN would increase the Stim1 Ca^2+^-sensitivity the >tenfold necessary to reverse SOCE; furthermore, the effects of GPN upon the EF-hand mutant (D76A) form of Stim1 that does not bind Ca^2+^, even up to 10 mM (Gudlur et al., 2018; Liou et al., 2005) (Fig. 6), implies that this inhibition of SOCE by GPN is not mediated by enhancing luminal Ca^2+^-binding to Stim1. We cannot formally exclude the possibility that GPN mimics Ca^2+^ binding, but this would have to be at a site distinct from the mutated EF-hand, so we currently consider Stim1 Ca^2+^-sensing as an unlikely target for lysosomal agents.

Stim1 puncta formation is a hallmark of the SOCE pathway and occurred as expected with all EYFP-Stim1 variants tested. Consistent with their inhibition of SOCE, GPN and nigericin dispersed Stim1 puncta (Fig. 6). Most remarkably, GPN even reversed the constitutive oligomers formed by the Stim1 EF-hand mutant (D76A) (Fig. 6). Kinetically, the rapid dispersal of Stim1 puncta by small molecules is consistent with the rapid inhibition of Ca^2+^ entry. This reversal of Stim1 puncta can readily explain the inhibition of SOCE and has been seen previously with other agents, such as 2-APB and its analogues (Dehaven et al., 2008; Goto et al., 2010) or the kinase inhibitor ML-9 (Smyth et al., 2008). Indeed, it is remarkable that the effects of GPN and ML-9 are almost identical in that they both inhibit SOCE, both disperse Stim1 puncta (even of a constitutively active EF-hand mutant), and both are less effective when Stim1 is overexpressed (see below). Nevertheless, the fact that another lysosomotropic agent, LLOMe, appeared to have no effect upon Stim1 puncta implies that this is not always observed with lysosomotropic agents. It is currently unclear why LLOMe is less efficient, but it is worth commenting that its stimulation of Ca^2+^ release exhibits more cell-to-cell variability than does GPN or nigericin. It also illustrates that LLOMe must be inhibiting SOCE at another site(s).

The monitoring of EYFP-Stim1 morphology clearly reveals that some lysosomal agents (GPN and nigericin) can reverse puncta and thereby inhibit SOCE. However, our data are somewhat paradoxical because we also show that the expression of EYFP-Stim1 variants renders SOCE less sensitive to inhibition by GPN (Fig. 6). Can we rationalize these results? Others suggest that overexpression of either Orai or Stim1 stabilizes the interaction between the two (Dehaven et al., 2008; Smyth et al., 2008) and this may explain why other puncta-disrupting agents (2-APB and ML-9) become less efficient inhibitors of Ca^2+^ entry (Dehaven et al., 2008; Smyth et al., 2008). We suggest the same occurs for GPN-mediated inhibition.

In addition to monitoring Stim1 activation (revealed by puncta assembly), we also investigated whether the downstream activation of Orai might also be affected. To activate Orai independently of ER Ca^2+^-release and Stim1 oligomerization, we activated Orai using a cytosolic fragment of Stim1 that contains most of the SOAR (Soboloff et al., 2012); we used the optogenetic variant, hBACCS2, in which SOAR is caged by LOV2-Jα (Ishii et al., 2015). Both GPN and LLOMe inhibited the Ca^2+^ entry evoked by hBACCS2 (Fig. 6). This suggests that these lysosomotropic agents interfere with SOAR-Orai interactions. The kinetics of GPN and LLOMe inhibition was faster than LOV2-Jα-SOAR reversal when the uncaging light was switched off (Fig. 6M), implying that GPN and LLOMe actively interfere with the stimulation of Orai and not with the hBACCS2 uncaging process. Of the two lysosomotropic agents, GPN was a more varied and generally slower inhibitor of the hBACCS2 response (Fig. 6M). However, it should be born in mind that when evoking SOCE with hBACCS2 (unlike SOCE with CPA) the ER Ca^2+^ stores are still replete so that GPN will not only inhibit Ca^2+^ entry evoked by hBACCS2 but also release Ca^2+^ from the stores (the latter increases cytosolic Ca^2+^, whereas the former blocks SOCE, so the slow fall is the net outcome of the two). LLOMe is not such a consistent stimulus of Ca^2+^ release, which is why its inhibition kinetics are not so masked. We hypothesise that lysosomotropic agents inhibit SOAR-Orai interactions, but it will require Stim-Orai intermolecular fluorescence resonance energy transfer studies (Derler et al., 2013) to further strengthen this.

GPN (but not the other agents) may also unexpectedly affect Orai1 activation by inducing its redistribution in the plasma membrane. As revealed with Orai1-EYFP, GPN promoted the formation of large dark patches within the Orai1 labelling (and indeed, this appeared to be a general effect upon inner-leaflet proteins as another lipid-anchored protein showed similar patches). The drop in fluorescence in these patches is either going to be due to quenching of the tag or to redistribution of the proteins. We consider it unlikely to be due to quenching by acidification when (1) the tags EYFP and TagRFP-T exhibit different sensitivities to pH (pK_a_ values 5.8 and 4.6, respectively); (2) the loss of fluorescence is so spatially restricted, as well as sustained; and (3) GPN acts as a weak base, not an acid (Atakpa et al., 2019). Although not formally proven, we hypothesise that membrane proteins facing the cytosol are redistributed by an unknown mechanism (e.g. occlusion by the insertion of other non-labelled proteins or structures that do not rapidly equilibrate). This would mean that Stim1 puncta formed across the cell will have a reduced likelihood of encountering Orai1 channels as the surface-area density has been reduced.

We do not consider lysosomal agents to be acting as simple Orai channel-pore blockers for multiple reasons. First, GPN only weakly inhibits SOCE in cells overexpressing Stim1 (Fig. 6), and hyper-expression does not normally prevent simple Orai pore blockade by agents such as 2-APB, La^3+^ or other organic blockers (Dehaven et al., 2008; Derler et al., 2013). Second, the SOCE inhibition by GPN is promptly reversed by the dynamin inhibitor dynasore (Fig. 8). The effect of dynasore is unlikely to be a dynamin-dependent event because it was not mimicked by genetically manipulating dynamin activity (Fig. 8), and probably adds to the growing list of off-site targets for this drug (Basagiannis et al., 2017; Preta et al., 2015). Regardless of its precise mechanism, dynasore would not be expected to reverse a pore blocker (particularly when dynasore itself can block entry when the order of addition is changed, via an unknown mechanism – Fig. 8). Together, the data are incompatible with simple pore blockade and suggest additional sites of action of lysosomotropic agents (summarized in Fig. 8G).

### A role for lysosomes?

Our model proposes that lysosomal agents inhibit SOCE and this appears to be primarily at two loci: by interfering with the oligomerization of Stim1 (GPN and nigericin) and by interfering with Orai interactions with SOAR (GPN and LLOMe). Are these effects dependent on their shared action at lysosomes or are these merely off-site effects? Unfortunately, our data do not definitively answer this key question. In favour of a primary lysosomal target, the three chemically unrelated lysosomal agents inhibit SOCE at concentrations that manifestly affect lysosomes. It is intriguing that the so-called Stim1 inhibitor ML-9 has also emerged as a lysosomotropic agent (Kondratskyi et al., 2014; Shaikh et al., 2018), with effects that strikingly mirror our GPN data. It is tempting to speculate that these four lysosomal agents act via lysosomes, but if these organelles are indeed involved, our other data limit the pathway by which this could occur, as we will briefly discuss.

First, we found that clustering lysosomes at the MTOC or periphery did not alter SOCE or the inhibition by lysosomotropic agents. The effect of repositioning contrasts with HeLa cells, in which shifting lysosomes to the periphery inhibited SOCE by Ca^2+^-buffering effects (Sbano et al., 2017). Our data suggest that if lysosomes are mediating the effects of the agents, this is independent of lysosomal placement (and might, for example, require a diffusible factor).

Second, these lysosomal agents increase lysosomal pH_L_. However, inhibition of the lysosomal H^+^-pump with bafilomycin A1 likewise increased lysosomal pH_L_ but did not mimic the other agents, in that it did not inhibit SOCE. Furthermore, GPN could still inhibit SOCE in the presence of bafilomycin A1 (Fig. 4). Together, it is therefore unlikely that these lysosomal agents inhibit SOCE only by increasing lysosomal pH_L_. Is it possible that LMP (and/or membrane repair pathways) regulates SOCE? There is indeed a correlation between LMP and the inhibition of SOCE since LLOMe, GPN and nigericin all evoke LMP (Atakpa et al., 2019; Heid et al., 2013; Katsnelson et al., 2016; Morgan et al., 2020; Repnik et al., 2017), whereas bafilomycin A1 does not (Repnik et al., 2017), and even inhibits LMP (Boya et al., 2003; Jessop et al., 2017). However, it will require further work to see whether LMP/membrane-repair regulates SOCE, and goes against LMP actually requiring Ca^2+^ influx for inflammasome signalling (Katsnelson et al., 2016). In summary, our data show that multiple lysosomal agents that can induce LMP inhibit SOCE, but a role for lysosomes is unclear.

### Ionic action?

If these agents are not acting via their common action at lysosomes, how might they be acting? We discount the trivial explanation of pH on multiple grounds. GPN was recently shown to act as a weak base that elevates the cytosolic pH (Atakpa et al., 2019). However, alkalinization of the cytosol is unlikely to explain the inhibition of SOCE because (1) the pH increase is transient, whereas the inhibition of SOCE is sustained; (2) nigericin and GPN have opposite effects upon pH but a common inhibition of SOCE; and (3) cytosolic alkalinization tends to increase SOCE and promote Stim1-Orai1 interactions (Gavriliouk et al., 2017; Mancarella et al., 2011; Tsujikawa et al., 2015).

Can lysosomal agents be acting by releasing lysosomal Ca^2+^? We have not explicitly addressed this because our studies predominantly use Ca^2+^ as a readout, so we cannot clamp cytosolic Ca^2+^ and monitor SOCE; Ca^2+^-dependent inhibition would need to be investigated electrophysiologically. The lack of effect of bafilomycin A1 upon GPN has been used to argue against its releasing Ca^2+^ from lysosomes (Atakpa et al., 2019) but, as we recently discussed (Morgan et al., 2020), this assumes that bafilomycin A1 induces a robust Ca^2+^ leak from lysosomal Ca^2+^ stores and this might not always be the case (Garrity et al., 2016). Furthermore, a transient Ca^2+^ release from lysosomes by lysosomal agents would have to induce a prolonged inhibition of SOCE and this seems kinetically incompatible. We currently consider it unlikely that this is a simple Ca^2+^-release phenomenon.

### A universal effect?

With such a robust inhibition of SOCE by these agents, has this been observed before? That GPN per se fails to evoke SOCE has indeed been suggested (Haller et al., 1996; Hui et al., 2015). Otherwise, many Ca^2+^ recordings in response to lysosomotropic agents, such as GPN, have, ironically, been conducted in Ca^2+^-free medium to isolate the intracellular Ca^2+^-release phase. Nevertheless, even those conducted in Ca^2+^-containing media, in which cytosolic Ca^2+^ spiking runs down, could have been misinterpreted as a depletion of the finite lysosomal store; indeed, it was only our monitoring of ER Ca^2+^ that revealed this issue. In view of the effect, it warrants interpretational caution for the acute inhibition of Ca^2+^ spiking by lysosomal agents unless multiple approaches are compared (Menteyne et al., 2006; Yamasaki et al., 2004).

In summary, we have shown that Ca^2+^ responses to lysosomal agents run down, even in Ca^2+^-containing media because the ER Ca^2+^-store becomes depleted, and the normal accompanying process of SOCE is inhibited (mostly by effects on Stim1 signalling). Application of these lysosomal agents should take into account these other potential consequences for the ER and SOCE.

## MATERIALS AND METHODS

### Cell culture

Cos-7 cells were a generous gift from Prof. Colin Akerman (University of Oxford, UK), cultured in Dulbecco's modified Eagle's medium containing 10% fetal calf serum, 2 mM glutamine, 100 U/ml penicillin and 100 µg/ml streptomycin at 37°C in 5% CO_2_. Cells were periodically treated with mycoplasma removal agent. For imaging, cells were either subcultured onto 25-mm diameter no. 1 glass coverslips or into ten-well Cellview Dishes (Greiner).

### Microscopy

Unless otherwise stated, cells were loaded, maintained and used in extracellular medium (ECM; 121 mM NaCl, 5.4 mM KCl, 0.8 mM MgCl_2_, 1.8 mM CaCl_2_, 6 mM NaHCO_3_, 25 mM HEPES and 10 mM Glucose, pH 7.4) at room temperature. To examine the effect of ECM with high K^+^, immediately before imaging, cells were washed three times and maintained in High-K^+^ ECM (5 mM NaCl, 145 mM KCl, 1 mM MgCl_2_, 1.8 mM CaCl_2_, 10 mM HEPES, and 10 mM Glucose, pH 7.4). Cells were imaged using a Nikon A1R laser-scanning confocal microscope equipped with 20×, 40× and 60× objectives, and used in Galvano mode to collect an image collected every 3-5 s. Multi-channel images were collected in channel-series mode (to reduce bleed through). The following standard blue, green, red and far-red spectral configurations were used unless otherwise stated (excitation/emission): 405 nm/450 nm, 488 nm/525 nm, 561 nm/595 nm and 647 nm/700 nm; all emission bandwidths are 50 nm except the 700 nm (75 nm). All images are single confocal sections.

### Monitoring Ca^2+^

Cytosolic Ca^2+^ was routinely monitored with intensimetric GCaMP6s (excitation/emission, 488/525 nm). For ratiometric cytosolic Ca^2+^ recordings, GEM-GECO1 was used, with cells excited at 405 nm and dual emissions recorded at 450/525 nm, the emission ratio being directly proportional to Ca^2+^. Luminal ER [Ca^2+^] was monitored using R-CEPIA1er. Single-cell fluorescence data were analysed using custom-written Magipix software (Dr Ron Jacob, King's College London, UK).

### Monitoring pH

To monitor cytosolic pH semi-quantitatively, cells were loaded with 2 µM BCECF/AM plus 0.03% Pluronic F127 for 50 min at room temperature and imaged on a Nikon A1R confocal microscope using dual excitation (405 and 488 nm) and single emission (525±25 nm). Data are expressed as the 488/405 ratio, which is directly proportional to pH. Lysosomal pH was qualitatively assessed by loading cells with 300 nM Lysotracker Red for 5 min at room temperature.

### Transfection and plasmids

After 1-3 days, when cells were at 50-70% confluency, they were transfected for 4-6 h with various plasmids using the transfection reagent JetPEI (Polyplus Transfection) in a ratio of 1 µg DNA to 2.0-2.5 µl of JetPEI. Cells were washed and used 16-24 h later.

The following plasmids were obtained as gifts from these authors via Addgene: GCaMP6s (Douglas Kim and the GENIE Project, 40753) (Chen et al., 2013); R-CEPIA1er (Masamitsu Iino, 58216) (Suzuki et al., 2014); the following from Tobias Meyer (Liou et al., 2005), SP-YFP-STIM1(23-685) (18857), SP-YFP-STIM1(D76A) (18859) and YFP-STIM1-deltaK (18861); GEM-GECO1 (Robert Campbell, 32442) (Zhao et al., 2011); hBACCS2-IRES-GFP (Takao Nakata, 72891) (Ishii et al., 2015); mTagRFP-Membrane-1 (Michael Davidson, 57992); Orai1-YFP (Anjana Rao, 19756) (Prakriya et al., 2006); K44A HA-dynamin 2 (Sandra Schmid, 34685); tdTomato-BicD2-FKBP12 (64205) or KIF5C-tdTomato-FKBP12 (64211), both from Gary Banker (Bentley et al., 2015); GPI-EGFP was a generous gift from Sergio Grinstein (Hospital for Sick Children, Toronto, ON, Canada); LAMP1-ECFP-FRB* was a generous gift from Takanari Inoue (Johns Hopkins School of Medicine, Baltimore, MD, USA).

### Reagents

GPN was obtained from Santa Cruz Biotechnology or Abcam. LLOMe was purchased from Cayman Chemical or Merck. CPA was from obtained from Merck. Dynasore was purchased from Abcam. AP21967 (rapalog) was obtained from Takara Bio. Lysotracker Red, Lysotracker Deep Red and BCECF/AM were obtained from Life Technologies. JetPEI was sourced from Polyplus Transfection. Mycoplasma removal agent was purchased from Bio-Rad. All other reagents were obtained from Sigma-Aldrich.

### Data analysis

All morphological analyses were conducted using Nikon NIS-Elements software (version 4).

#### Lysosomal translocation

The rapalog-induced repositioning of lysosomes was assessed by quantifying lysosomal fluorescence along a line (5-pixel width) drawn between the nucleus and plasma membrane, i.e. a profile intensity plot. Fluorescence at each point was then expressed as a percentage of the total mean fluorescence along the line. Because cells are different sizes, the nuclear-plasmalemmal distance was normalized to 100% and the mean fluorescence binned from each 5%-distance increment to allow data to be collated from different cells. Significance compared to the control was determined by paired *t*-tests in GraphPad Prism 6 using the Holm–Sidak method (alpha=5% and not assuming a constant s.d.).

#### Stim1 morphological changes

To quantify Stim1-puncta formation and reversal, the size and shape of YFP-Stim1 structures were determined using threshold masking. Images were first smoothed to remove noise, and the threshold-mask settings of the single cell were defined using an image of stable CPA-induced puncta. These settings were then propagated through every frame of the time series. The binary-mask properties were exported from single images for each condition: basal, CPA plateau and 2-5 min after addition of the lysosomal agent. Multiple structures were detected per cell, and these were averaged to give single-cell means for each parameter (area, perimeter, length and circularity).

#### Plasma membrane morphological changes

To quantify patch size in the plasma membrane marker, images were first smoothed to remove noise and the single-cell boundary was drawn to first determine the total cell area. To determine the patch size, a threshold mask selected the non-patch plasmalemmal fluorescence and this mask was inverted to select non-fluorescent regions; by generating the intersection with the single-cell boundary, the area of the patches was quantified and expressed as a percentage of the total cell area.

When simultaneously monitoring plasma membrane remodelling (inner- and outer-leaflet) and Ca^2+^ signals (GEM-GECO1), multiple regions of interest (ROI) were drawn manually, one per contiguous plasma membrane patch, and fluorescence monitored over time. For each ROI, the amplitude (ΔF/F_0_) and lag time were determined (lag=the time between agent addition and the first point of deviation from F_0_). These ROI values were then averaged per cell to give a single-cell mean.

### Statistical analyses

Statistics were determined either using GraphPad Prism 6 or GraphPad Instat 3.1. Two data sets were compared using a two-tailed Student's *t*-test, whereas a one-way ANOVA and Tukey–Kramer (or Bonferroni) post-hoc test were used for three or more conditions. For concentration-response curves, Dunn's multiple comparisons test compared different concentrations with the vehicle control. Data were paired where appropriate. Normality was determined using the Kolmogorov and Smirnov test, and non-parametric tests were applied when data failed normality. Experiments were conducted on at least three separate cell preparations on different days, with multiple transfection replicates per condition. Data throughout are expressed as the mean±s.e.m. of *n* cells (from *N* different experiments).

## Supplementary Material

Supplementary information

Reviewer comments
