# Supplementary material for: Lysosomal agents inhibit store-operated Ca2+ entry
Source: J Cell Sci. 2021 Jan 27;134(2):jcs248658. doi: 10.1242/jcs.248658 (PMC7860125; doi:10.1242/jcs.248658)
Supplement: Supplementary information [file joces-134-248658-s1.pdf]

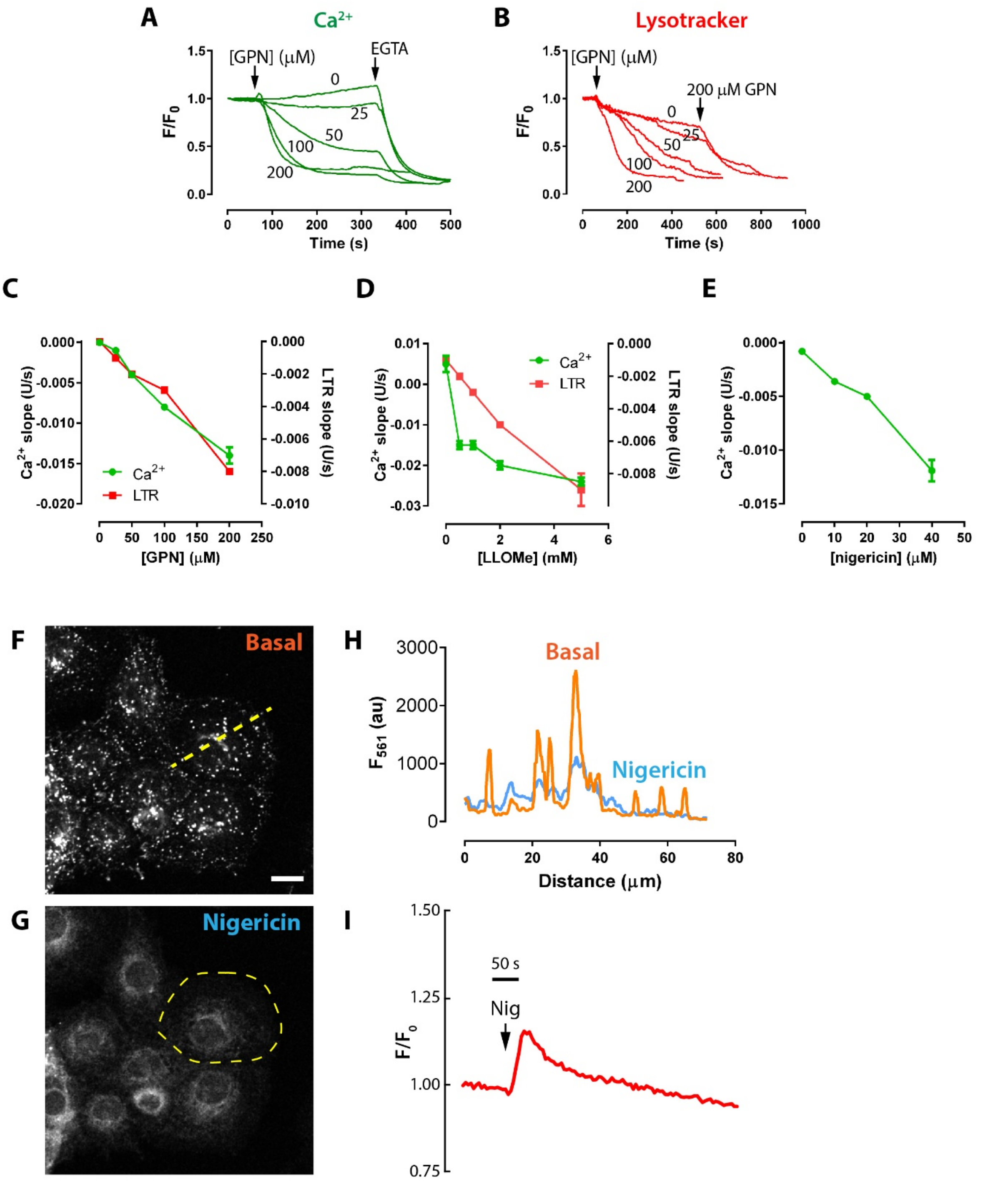

Figure S1. Effect of lysosomal agents on Ca^{2+} entry and Lysotracker Red labelling.
Concentration-dependence of the lysosomal agents with respect to SOCE and lysosomes; cells labelled with GCaMP6s/Lysotracker Red were preincubated with 50 µM CPA for ≥20 min and the indicated concentrations of agents added. Illustrative traces of the concentration-dependence of GPN with respect to SOCE (A) and Lysotracker Red (B). (C-E) Collated *kinetics* of the decrease in SOCE (Ca^{2+}) or Lysotracker Red (LTR) as determined simultaneously (the same experiments as **Figure 4**E-G, n = 82-303, N = 3-4). Kinetics were quantified by a linear regression of the initial decrease (traces were first normalized to the initial fluorescence, F_0). (F-I) Nigericin (20 µM) induces a translocation of

Journal of Cell Science • Supplementary information

LTR from lysosomes to other endomembranes. Images of a field of cells labelled with LTR prior to (Basal, F) and after nigericin addition (G). (H) Corresponding profile plots of fluorescence along the dotted yellow line in F (and also applied to G). (I) Mean whole-cell fluorescence of the single cell outlined in G. Scale bar = 20 µm. Representative of ≥6 experiments.

Journal of Cell Science • Supplementary information

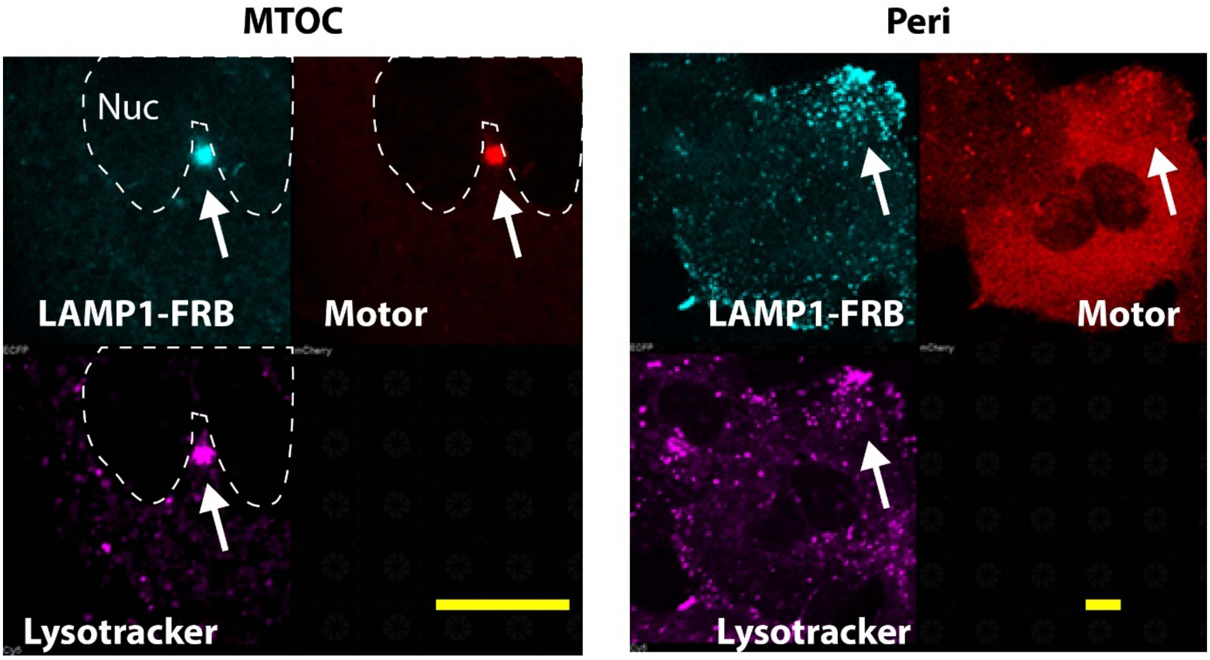

Figure S2. Rapalog-induced translocation of lysosomes indicated by LAMP1

Data accompany main **Figure 5**. Single-cell colocalization of LAMP1-ECFP-FRB (*cyan*), motor-binding proteins (*red* — tdTomato-BicD2-FKBP12 [MTOC] or KIF5C-tdTomato-FKBP12 [Peri]) and Lysotracker Deep Red (*magenta*). Nucleus = Nuc. Scale bars = 10 µm.

Journal of Cell Science • Supplementary information

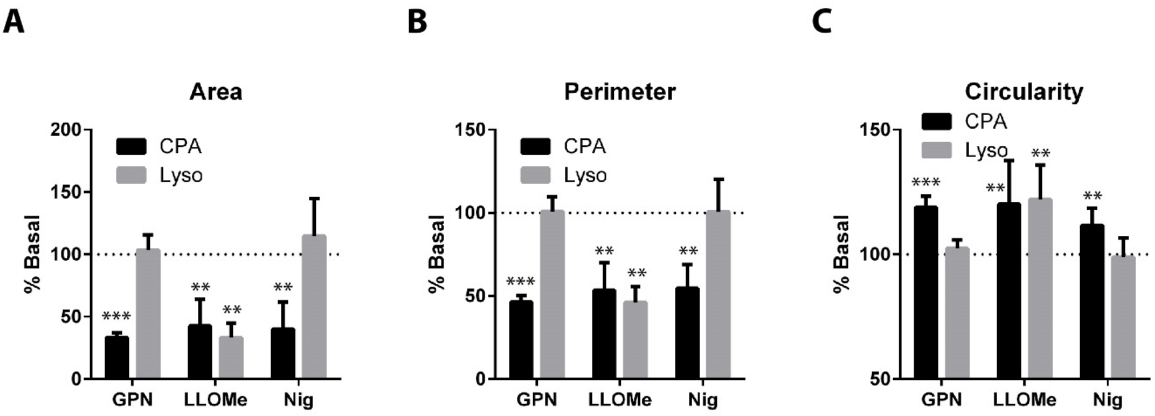

Figure S3. Stim1 Morphology Changes

Measurements of the cell-average area, perimeter and circularity of Stim1-positive structures in the same experiments of **Figure 6** determined using binary-threshold masking. In resting (pre-CPA) cells, the raw values were: 45 ± 19 μm^2 (area), 29 ± 6 µm (perimeter), 0.67 ± 0.02 (circularity). **P<0.01, ***P<0.001, paired, repeated measures of ANOVA versus *Basal*.
