# Supplementary material for: Lysosomal agents inhibit store-operated Ca2+ entry
Source: J Cell Sci. 2021 Jan 27;134(2):jcs248658. doi: 10.1242/jcs.248658 (PMC7860125; doi:10.1242/jcs.248658)
Supplement: Reviewer comments [file joces-134-248658_review_history.pdf]

Lysosomal agents inhibit store-operated Ca^{2+} entry

Anthony Morgan and Antony Galione
DOI: 10.1242/jcs.248658

Editor: John Heath

Review timeline

Original submission:	11 May 2020
Editorial decision:	6 July 2020
First revision received:	9 November 2020
Editorial decision:	7 December 2020
Second revision received:	8 December 2020
Accepted:	9 December 2020

Original submission

First decision letter

MS ID#: JOCES/2020/248658

MS TITLE: Lysosomotropic agents inhibit store-operated Ca^{2+} entry

AUTHORS: Anthony Morgan and Antony Galione
ARTICLE TYPE: Research Article

We have now reached a decision on the above manuscript.

To see the reviewers' reports and a copy of this decision letter, please go to: https://submit-jcs.biologists.org and click on the 'Manuscripts with Decisions' queue in the Author Area. (Corresponding author only has access to reviews.)

As you will see, the reviewers raise a number of substantial criticisms that prevent me from accepting the paper at this stage. They suggest, however, that a revised version might prove acceptable, if you can address their concerns. In particular, one referee states ' the most important critique in my review are the comparisons between the drugs dose-response in affecting lysosomal function (these are available or can be inferred from previous publications) and inhibiting SOCE -- needs to be measured by the authors'.

If you think that you can deal satisfactorily with the criticisms on revision, I would be pleased to see a revised manuscript. We would then return it to the reviewers.

We are aware that you may currently be unable to access the lab to undertake experimental revisions. If it would be helpful, we encourage you to contact us to discuss your revision in greater detail. Please send us a point-by-point response indicating where you are able to address concerns raised (either experimentally or by changes to the text) and where you will not be able to do so within the normal timeframe of a revision. We will then provide further guidance. Please also note that we are happy to extend revision timeframes as necessary.

Please ensure that you clearly highlight all changes made in the revised manuscript. Please avoid using 'Tracked changes' in Word files as these are lost in PDF conversion.

© 2020. Published by The Company of Biologists under the terms of the Creative Commons Attribution License (https://creativecommons.org/licenses/by/4.0/).

I should be grateful if you would also provide a point-by-point response detailing how you have dealt with the points raised by the reviewers in the 'Response to Reviewers' box. Please attend to all of the reviewers' comments. If you do not agree with any of their criticisms or suggestions please explain clearly why this is so.

Reviewer 1

Advance summary and potential significance to field

The authors, in text that is not always carefully written, present interesting observations (captured most clearly in Fig. 3D and E), derived from applying a variety of approaches. Namely, some agents (GPN, LLOMe and nigericin) appear to uncouple store-depletion from activation of store-operated Ca2+ entry (SOCE). But no clear story emerges. Not all perturbations of lysosomes inhibit SOCE (consistent with published work), but nor are there consistent effects of agents across assays: LLOMe, for example, inhibits SOCE, but not STIM1 clustering, while other agents do both; and while GPN is claimed to reverse clustering of STIM1ΔK, it does not inhibit the SOCE evoked by it; optogenetic activation of SOCE (which does not require STIM1 clustering) is also inhibited by GPN. I am not disputing these observations, so much as expressing concern that until a clearer explanation emerges, the work is likely to be of limited interest. Furthermore, not all conclusions are justified by the results presented. In summary, the work does not provide a sufficiently coherent or well-developed explanation of SOCE inhibition to justify publication in J. Cell Sci.

Comments for the author

Major

1. Given the key conclusions, it is important to see a direct comparison of store-depletion evoked by CPA in the absence and presence of extracellular Ca2+: the difference tells us what SOCE should look like when stores are emptied.
2. Text comparisons of Fig. 2E and G: it is incorrect to compare rates of uncalibrated fluorescence traces. Neither cytosolic [Ca2+] (measured with a high-affinity sensor that is almost certainly responding non-linearly to [Ca2+]c) nor pH are calibrated. Furthermore, even if [Ca2+] and [H+] were calibrated, it would be an inapt comparison that use the log of once concentration (pH) and not of the other. I don't think these flaws detract from the argument that nigericin is probably acting internally, but the comparisons of rates should be either removed or validated. The same applies to 'kinetics' reported in Fig. 6L. Text in Methods should also be more carefully phrased to avoid implying that fluorescence is directly related to underlying Ca2+ or pH.
3. The legend to Fig. 1 needs to be completed so that each panel is identified (presently only A). If non-parametric tests are used for analysis, it would be more appropriate to present data non-parametrically (eg, median and ranges). No statistics reported in 1C. Fig. 2: panels C, F and I are not described, nor is their statistical analysis. Presumably Fig. 8C is in Ca2+-free medium. All legends require attention to ensure that each panel in each figure is described and all statistics are reported.
4. What exactly is shown in Fig. 5A? Are these single confocal sections or stacks for Lysotracker? The strong impression is of loss of lysosomes (or loss of their ability to report their presence by low pH), as much as redistribution.
5. Fig. 6: the STIM1 images are unconvincing. The description in the text (p8, para 2) is not justified by the images shown. The 'puncta dispersal index' (B and C) should be described in sufficient detail to know how it was calculated (Methods need expanding). The summary data (6F) appears to make no allowance for the decline in fluorescence with time.
6. The results shown in Fig. 7 are intriguing, but they require more thorough analysis (quantification, rather than a representative image) and explanation. The results with dynasore (Fig. 8) are also intriguing (if a little off the beaten track), but they lack sufficient controls, and the curious order-of-addition observations lack any convincing explanation. Could GPN and dynasore bind to each other? Where is the evidence that the effects are a reversal of GPN effects, rather than a stimulation of an increase in [Ca2+]c? Are the effects clearly on SOCE?

Minor

1. The title captures only part of the story, since nigericin is not a lysosomotropic agent.

© 2020. Published by The Company of Biologists under the terms of the Creative Commons Attribution License (https://creativecommons.org/licenses/by/4.0/).

2. Throughout, the text needs careful revision to correct for missing/incorrect words, sloppy prose (eg p6, line 10 'mobilized the ER'), etc.
3. Several methods require fuller descriptions: microscopy filters, etc; correction of the mistaken impression that indicators directly report pH of [Ca2+]; image analysis methods; sources of all materials (eg JetPEI).
4. For the non-specialist reader, it might be helpful to elaborate further on BACCS2.
5. Given that 'CAD' is also used to describe the activating domain of STIM1, it might be better to use another abbreviation for cationic amphiphilic drugs (perhaps amphiphilic cationic drugs, ACD).
6. Notwithstanding the complexity of the phenomena observed, the Discussion is too long. Statement (a) on p12, para 2 is incorrect (see Fig. 1A).

Reviewer 2

Advance summary and potential significance to field

The manuscript explores the interesting new finding that three pharmacological agents commonly used to probe the function of lysosomes: GPN and LLOMe (permeabilize lysosomal membranes) and Nigericin (K+/H+ ionophore) have as side effect blocking the store-operated calcium entry (SOCE) in Cos-7 cells. In contrast, Baf A1, an inhibitor of the v-ATPase that acidifies endo-lysosomes, had no effect on SOCE. The main results are based on a powerful combination of genetically-encoded calcium indicators that allow the authors to monitor simultaneously cytosolic and ER calcium in response to different pharmacological treatments or physiological agonists. The manuscript uses a combination of techniques to explore in depth the effect of the inhibitors on the two components of the SOCE, STIM1 and ORAI1.
The effect of these particular lysosomotropic agents on SOCE is intriguing and relevant because the function of lysosomes is often investigated using pharmacological agents, including the ones described in the manuscript. The findings in the manuscript prompt a reevaluation of the previous data based on these inhibitors, especially data related to calcium release or uptake by endolysosomal compartments, results that could be confounded by the side effects of the inhibitors.

Comments for the author

Although the manuscript and the results are interesting and mostly convincing there are a few weaknesses that need to be addressed.
- The three pharmacological inhibitors investigated in the study are used at one concentration throughout the manuscript. Why the respective concentrations were chosen and how do they compare to other studies that use the compounds? It would be particularly relevant to know if the effect on SOCE is dose dependent and how the SOCE inhibition dose dependence compares to the effect on lysosomes – this finding would help understand if there is a range of concentrations where the drugs can be used for their effect on lysosomes without affecting SOCE.
- The conclusions based on imaging data are not convincing. Most of the imaging data is not quantitatively analyzed (or the analysis is not described, as in Fig 6B and 6C, 7H) and the effects described in the text are not readily apparent. This is the case for Figs 4, 5, and 6.

-- Why is Fig 5 relevant to the study? It uses a great technique, but there is no quantitative analysis of the data and the relevance for the current study is not clear
-- The change in puncta distribution shown in 6B and 6C are not at all apparent in the images in 6A. Calibration bars are also missing.
-- I found Fig. 7 particularly weak: the control for ORAI1 distribution and dark patches formation in response to a physiological stimulus is missing. The formation of the dark patches is not quantified and their size is difficult to estimate without calibration bars in most of the images.
-- One could interpret the data as the cells being unhealthy, as supported by the traces in 7H, where calcium responses cease before the black patches start forming
– although the results are based on traces from a single cell and the image analysis is not described.

The manuscript is missing a lot of details in terms of methodology and data analysis that make the interpretation of the results difficult. These need to be added.

© 2020. Published by The Company of Biologists under the terms of the Creative Commons Attribution License (https://creativecommons.org/licenses/by/4.0/).

o Why were Cos-7 used for the study? In the manuscript there is no mention of what cells were used until page 7.
o The number of independent experiments from which the cells were analyzed is not mentioned for any of the results.
o How and when was the high K+ the solutions added in 2E, 2G, 4D?
o How much ATP was used in 1A?
o When was the light turned off in 6G, K, L?
o Why was BafA1 added chronically (30 min)? This is not commonly used and the relevance is not clear for this study.

First revision

Author response to reviewers' comments

Reviewer 1 Comments for the Author
Major
1. Given the key conclusions, it is important to see a direct comparison of store-depletion evoked by CPA in the absence and presence of extracellular Ca2+: the difference tells us what SOCE should look like when stores are emptied.

New data presented (Figs 1D-F), thank you for the suggestion.

2. Text comparisons of Fig. 2E and G: it is incorrect to compare rates of uncalibrated fluorescence traces. Neither cytosolic [Ca2+] (measured with a high-affinity sensor that is almost certainly responding non-linearly to [Ca2+]c) nor pH are calibrated. Furthermore, even if [Ca2+] and [H+] were calibrated, it would be an inapt comparison that use the log of once concentration (pH) and not of the other. I don’t think these flaws detract from the argument that nigericin is probably acting internally, but the comparisons of rates should be either removed or validated. The same applies to ‘kinetics’ reported in Fig. 6L. Text in Methods should also be more carefully phrased to avoid implying that fluorescence is directly related to underlying Ca2+ or pH.

We entirely agree with the referee that the reporter responses are, by definition, non-linear. However, there is a substantial literature on the pitfalls and artifacts of *in situ* calibrations for intensimetric reporters, and therefore we do not to calibrate to avoid introducing such errors.

We do not feel that our results are at all negated by this because the reporters are acting within the quasi-linear range. We have not actually ‘compared rates’ between pH and Ca^{2+}, other than the crude observation that the time-to-peak of the changes are self-evidently very different.

This point aside, we consider it useful to have a semi-quantitative analysis of uncalibrated data (to illustrate reproducibility and differences between agents), even for the kinetics comparisons in Fig 6M. This clearly shows there are differences between the conditions, but we have been careful not to claim that these qualitative data correspond proportionally to *absolute* rates of Ca^{2+} removal or Ca^{2+} flux rates.

3. The legend to Fig. 1 needs to be completed so that each panel is identified (presently only A). If non- parametric tests are used for analysis, it would be more appropriate to present data non-parametrically (eg, median and ranges). No statistics reported in 1C. Fig. 2: panels C, F and I are not described, nor is their statistical analysis. Presumably Fig. 8C is in Ca2+-free medium. All legends require attention to ensure that each panel in each figure is described and all statistics are reported.

We apologise for any oversights and lack of detail and statistics. These are now addressed in the Methods, Figures and all Legends.

Fig 8C is in Ca^{2+}-containing medium, as indicated (the GPN response is always transient in Ca^{2+}-

© 2020. Published by The Company of Biologists under the terms of the Creative Commons Attribution License (https://creativecommons.org/licenses/by/4.0/).

containing as it is in Figs. 2,3,4,7).

4. What exactly is shown in Fig. 5A? Are these single confocal sections or stacks for Lysotracker? The strong impression is of loss of lysosomes (or loss of their ability to report their presence by low pH), as much as redistribution.

These are single sections as the cells are so flat and thin that z-stacks gain little (now noted in Microscopy Methods). As indicated in the original paper [1], association of lysosomes with molecular motors results in vesicular redistribution to the MTOC or periphery – hence rapalog induces colocalization of the lysosomes (cyan) and motor-binding proteins (red), as indicated by the arrows.

This is not due to loss of lysosomes (or Lysotracker labelling) but their physical 'clearance' from across the cell. We see the same redistribution pattern with the LAMP1-FRB 'bait' (which is independent of lysosomal pH and Lysotracker-loading). We have re-worded the text to clarify, and include a new Figure S2 with LAMP1.

5. Fig. 6: the STIM1 images are unconvincing. The description in the text (p8, para 2) is not justified by the images shown. The 'puncta dispersal index' (B and C) should be described in sufficient detail to know how it was calculated (Methods need expanding). The summary data (6F) appears to make no allowance for the decline in fluorescence with time.

We apologise for the unconvincing images, in part due to the low magnification. We now include (a) higher magnification images and (b) a new quantitation analysis of Stim1 morphology (based on threshold masks – see Methods). The new figure depicts the *length* of Stim1 structures, but the new Supplemental Figure S3 also includes other parameters such as area, perimeter and circularity.

6. The results shown in Fig. 7 are intriguing, but they require more thorough analysis (quantification, rather than a representative image) and explanation. The results with dynasore (Fig. 8) are also intriguing (if a little off the beaten track), but they lack sufficient controls, and the curious order-of-addition observations lack any convincing explanation. Could GPN and dynasore bind to each other? Where is the evidence that the effects are a reversal of GPN effects, rather than a stimulation of an increase in [Ca2+]c? Are the effects clearly on SOCE?

Fig. 7. We apologise for lack of quantitation of the plasma-membrane morphology changes. We have now addressed this by including (a) the Area dimensions of the patches induced by different conditions; (b) fluorescence changes (and their *timings* relative to Ca^{2+} responses) in the experiments with dual-labelling of the inner- and outer-leaflet.

Fig. 8. The GPN and dynasore experiments are mainly included to provide another functional antagonism of the GPN effect (so as to minimize the likelihood of GPN's being a simple pore-blocker). We cannot, unfortunately, provide a mechanism.

- As for GPN and dynasore binding to one another (and inactivating), we consider this improbable when GPN still acts when added *after* dynasore (Figs. 8B, 8C) or vice versa.
- This can *only* be due to a restoration of SOCE and the reversal of GPN because:
  - The intracellular Ca^{2+} stores are completely emptied by CPA (Figs. 1).
  - The effects are clearly due to SOCE because subsequent addition of extracellular EGTA eliminates the Ca^{2+} response (Fig. 8B).

Minor
1. The title captures only part of the story, since nigericin is not a lysosomotropic agent.
Point taken and now changed, thank you.

2. Throughout, the text needs careful revision to correct for missing/incorrect words, sloppy prose (eg p6, line 10 'mobilized the ER'), etc.
We apologise for this and have made every effort to amend errors.

© 2020. Published by The Company of Biologists under the terms of the Creative Commons Attribution License (https://creativecommons.org/licenses/by/4.0/).

3. Several methods require fuller descriptions: microscopy filters, etc; correction of the mistaken impression that indicators directly report pH of [Ca2+]; image analysis methods; sources of all materials (eg JetPEI).
We apologise and now include excitation/emission details.

We did in fact include the JetPEI supplier (Polyplus) in the original methods section (bottom of original page 17). We have now moved this to the next page with the other reagents for consistency.

4. For the non-specialist reader, it might be helpful to elaborate further on BACCS2.
We have now included a cartoon (Fig. 6) and a clearer description.

5. Given that 'CAD' is also used to describe the activating domain of STIM1, it might be better to use another abbreviation for cationic amphiphilic drugs (perhaps amphiphilic cationic drugs, ACD).
Fair point. We have now removed this entirely.

6. Notwithstanding the complexity of the phenomena observed, the Discussion is too long. Statement (a) on p12, para 2 is incorrect (see Fig. 1A).
It is unclear what the referee means by the error in point (a)? We were discussing all reasons why SOCE could reverse, and store-refilling might be one obvious reason. Our data show that it this is not the explanation, and we state this a few sentences later.

The Discussion has been tightened up and shortened in places.

Reviewer 2 Comments for the Author:
Although the manuscript and the results are interesting and mostly convincing, there are a few weaknesses that need to be addressed.
•The three pharmacological inhibitors investigated in the study are used at one concentration throughout the manuscript. Why the respective concentrations were chosen and how do they compare to other studies that use the compounds? It would be particularly relevant to know if the effect on SOCE is dose dependent and how the SOCE inhibition dose dependence compares to the effect on lysosomes – this finding would help understand if there is a range of concentrations where the drugs can be used for their effect on lysosomes without affecting SOCE.

We apologise for not justifying the concentrations. We now include new data showing the concentration-response relationships of the agents against both SOCE and lysosomes (Lysotracker fluorescence) (**Figs. 4E-G and S1A- E**). Broadly speaking, each agent affects SOCE and lysosomes over similar ranges.

•The conclusions based on imaging data are not convincing. Most of the imaging data is not quantitatively analyzed (or the analysis is not described, as in Fig 6B and 6C, 7H) and the effects described in the text are not readily apparent. This is the case for Figs 4, 5, and 6.

We apologise for insufficient analysis and explanations. We now include new analyses of:
- Changes in Stim1 morphology using threshold masks to determine length, area, perimeter, circularity of Stim1-positive structures (Fig. 6).
- Bafilomycin A1 effect upon Lysotracker fluorescence (Fig. 4).
- Lysosome redistribution (Fig. 5).

-- Why is Fig 5 relevant to the study? It uses a great technique, but there is no quantitative analysis of the data and the relevance for the current study is not clear
We apologise if this was not clear. In the Discussion, we cited a recent study [2] where lysosomes altered SOCE; in that study, manipulating lysosomal positioning modulated this effect, presumably by altering local Ca^{2+} buffering around the channels by the lysosomes. Therefore, we mimicked this in our system to see if it likewise altered SOCE (and GPN).

- We have moved this reference (and rationale) to the Results and re-phrased this to hopefully clarify this point.
- We are confused by the statement about "no quantitation" when the responses were

© 2020. Published by The Company of Biologists under the terms of the Creative Commons Attribution License (https://creativecommons.org/licenses/by/4.0/).

fully analysed and presented in Fig 5F. We do now include a quantitation of the repositioning (**Fig. 5B**), in case that is what the referee meant.

-- The change in puncta distribution shown in 6B and 6C are not at all apparent in the images in 6A. Calibration bars are also missing.

We now include higher resolution, scaled micrographs to illustrate the point better.

-- I found Fig. 7 particularly weak: the control for ORAI1 distribution and dark patches formation in response to a physiological stimulus is missing. The formation of the dark patches is not quantified and their size is difficult to estimate without calibration bars in most of the images.

- We are sorry for the lack of quantitation, we now include new analyses of patch size and timing (relative to Ca^{2+}).
- We also now include a physiological stimulus (ATP via purinoceptors) plus new data with nigericin.

-- One could interpret the data as the cells being unhealthy, as supported by the traces in 7H, where calcium responses cease before the black patches start forming
- although the results are based on traces from a single cell and the image analysis is not described.
Responses are now quantified (Fig 7).
How does one define 'unhealthy'? Often, compromised cells exhibit an elevated [Ca^{2+}], and yet only discrete Ca^{2+} spikes are evoked that return to basal.
This concentration of GPN is commonly used by other laboratories and so is of relevance and interest to users.

The manuscript is missing a lot of details in terms of methodology and data analysis that make the interpretation of the results difficult. These need to be added.

Completed.

o Why were Cos-7 used for the study? In the manuscript there is no mention of what cells were used until page 7.
They are a commonly used model for Ca^{2+} signalling and lysosome biology and are easy to transfect. We now mention the cell type at the start of the Results.

oThe number of independent experiments from which the cells were analyzed is not mentioned for any of the results.
Apologies. Now added to the Methods and each Legend.

oHow and when was the high K+ the solutions added in 2E, 2G, 4D?
Added to Methods.

oHow much ATP was used in 1A?
100µM. Added to legend.

oWhen was the light turned off in 6G, K, L?
We assume the referee means the original 6H,I,K. We now replace arrows with a time bar to avoid ambiguity (new Figs. 6H-K).

oWhy was BafA1 added chronically (30 min)? This is not commonly used and the relevance is not clear for this study.
On the contrary, Bafilomycin A1 is commonly used to collapse the lysosomal pH as it a specific inhibitor of the V- ATPase (nearly 1000 papers on Pubmed).

The lysosomal pH gradient does not immediately collapse upon V-ATPase inhibition, it requires the luminal H^+ to leak out, and this leak rate can be slow (tens of minutes) [3], or even negligible in 'tighter' acidic compartments [4]. Hence, we used a longer incubation times, as do respected labs in the field e.g. [5-8].

© 2020. Published by The Company of Biologists under the terms of the Creative Commons Attribution License (https://creativecommons.org/licenses/by/4.0/).

The use of bafilomycin A1 is entirely appropriate to probe whether these agents exert their SOCE effect via lysosomes.

References

1 Bentley, M., Decker, H., Luisi, J. and Banker, G. (2015) A novel assay reveals preferential binding between Rabs, kinesins, and specific endosomal subpopulations. The Journal of cell biology. **208**, 273-281

2 Sbano, L., Bonora, M., Marchi, S., Baldassari, F., Medina, D. L., Ballabio, A., Giorgi, C. and Pinton, P. (2017) TFEB-mediated increase in peripheral lysosomes regulates store-operated calcium entry. Sci Rep. **7**, 40797

3 Atakpa, P., Thillaiappan, N. B., Mataragka, S., Prole, D. L. and Taylor, C. W. (2018) IP3 Receptors Preferentially Associate with ER-Lysosome Contact Sites and Selectively Deliver Ca^{2+} to Lysosomes. Cell Rep. **25**, 3180-3193 e3187

4 Morgan, A. J. and Galione, A. (2007) NAADP induces pH changes in the lumen of acidic Ca^{2+} stores. Biochem J. **402**, 301-310

5 Brailoiu, E., Hoard, J. L., Filipeanu, C. M., Brailoiu, G. C., Dun, S. L., Patel, S. and Dun, N. J. (2005) Nicotinic acid adenine dinucleotide phosphate potentiates neurite outgrowth. The Journal of biological chemistry. **280**, 5646-5650

6 Zhang, X., Cheng, X., Yu, L., Yang, J., Calvo, R., Patnaik, S., Hu, X., Gao, Q., Yang, M., Lawas, M., Delling, M., Marugan, J., Ferrer, M. and Xu, H. (2016) MCOLN1 is a ROS sensor in lysosomes that regulates autophagy. Nat Commun. **7**, 12109

7 Favia, A., Pafumi, I., Desideri, M., Padula, F., Montesano, C., Passeri, D., Nicoletti, C., Orlandi, A., Del Bufalo, D., Sergi, M., Ziparo, E., Palombi, F. and Filippini, A. (2016) NAADP-Dependent Ca^{2+} Signaling Controls Melanoma Progression, Metastatic Dissemination and Neoangiogenesis. Sci Rep. **6**, 18925

8 Willett, R., Martina, J. A., Zewe, J. P., Wills, R., Hammond, G. R. V. and Puertollano, R. (2017) TFEB regulates lysosomal positioning by modulating TMEM55B expression and JIP4 recruitment to lysosomes. Nat Commun. **8**, 1580

Second decision letter

MS ID#: JOCES/2020/248658

MS TITLE: Lysosomal agents inhibit store-operated Ca²⁺ entry

AUTHORS: Anthony Morgan and Antony Galione
ARTICLE TYPE: Research Article

We have now reached a decision on the above manuscript.

To see the reviewers' reports and a copy of this decision letter, please go to: https://submit-jcs.biologists.org and click on the 'Manuscripts with Decisions' queue in the Author Area. (Corresponding author only has access to reviews.)

As you will see, the reviewers gave favourable reports but raised some critical points that will require amendments to your manuscript. I hope that you will be able to carry these out, because I would like to be able to accept your paper.

We are aware that you may be experiencing disruption to the normal running of your lab that makes experimental revisions challenging.If it would be helpful, we encourage you to contact us to discuss your revision in greater detail. Please send us a point-by-point response indicating where you are able to address concerns raised (either experimentally or by changes to the text) and where you will not be able to do so within the normal timeframe of a revision. We will then

© 2020. Published by The Company of Biologists under the terms of the Creative Commons Attribution License (https://creativecommons.org/licenses/by/4.0/).

provide further guidance. Please also note that we are happy to extend revision timeframes as necessary.

Please ensure that you clearly highlight all changes made in the revised manuscript. Please avoid using 'Tracked changes' in Word files as these are lost in PDF conversion.

I should be grateful if you would also provide a point-by-point response detailing how you have dealt with the points raised by the reviewers in the 'Response to Reviewers' box. Please attend to all of the reviewers' comments. If you do not agree with any of their criticisms or suggestions please explain clearly why this is so.

Reviewer 1

Advance summary and potential significance to field

Provided in first review

Comments for the author

The authors have responded to earlier comments, but incompletely.
Major original points:
1. The new data in Fig.1D-F helpfully indicate the normal scale of SOCE. OK 2. It seems incorrect to suggest that all reporters are acting within the 'quasi-linear' range. In the new panels (Fig 1D,E), for example, the response of GCaMP6S to CPA matches that produced by ionomycin (which very likely saturates the indicator). Furthermore, the F/F0 reaches about 35. Since the reported dynamic range of the indicator is ~60, and it is likely to be ~half-saturated at resting cytosolic [Ca2+] (F0), this too suggests saturation. Notwithstanding the limitations of calibrating fluorescence signals, I do not agree that the need to consider non-linear responses of indicators has been adequately addressed. The issue has clear implications. For example, the authors attach importance to the suggestion that the rate of change of pH and Ca2+ differ (p5), but that argument is not sustainable without taking account of the non-linear relationship between fluorescence and analyte.
3. OK 4. The legend to Fig. 5A remains unclear: although I figured it out, it should state absolutely clearly which panels show rapolog and which FKBP proteins. I still do not see, from these panels, evidently all from thin sections that include most of the cell, where all the staining in control goes to after rapalog treatment. There is demonstrably vastly more staining in Ctrl then in either of the other panels. I don't think that either the revised text or Fig. S2 effectively addresses the issue. Why is there such a loss of LysoTracker staining after the rapalog treatments?
5. In quantifying responses of STIM1 in Fig. 6, the authors use STIM1 length. It is not clear to me what this means. Is this an inverse reporter of puncta formation? I acknowledge that the other measures provided in Fig. S3 provide alternative supportive quantification. The first 3 columns (basal and CPA) of Fig. 6A are all effectively replicates (ie 3 basal, and 3 store-depleted cells), yet the within-column variability is as great as that between the columns. In short, I find the images in Fig. 6A unconvincing (they are more convincing in Fig. 6D)
6. OK on quantification. The observations in Fig 7 and 8 remain largely phenomenological, but that may be the nature of the beast at this stage.

Reviewer 2

Advance summary and potential significance to field

The manuscript shows that agents that alter lysosomal membrane integrity also cause Ca2+ release from ER and inhibition of the STIM/ORAI pathway.
Lysosomotropic agents are commonly used to permeabilize the lysosomal membrane in order to equilibrate the ionic concentrations between the cytosol and lysosomal lumen to study lysosomal function. This study shows that the results of experiments using such agents are not only due to lysosomal membrane permeabilization, but to a more complex effect on the cells. At the same time these agents cannot be used for experiments investigating cellular calcium signaling and

© 2020. Published by The Company of Biologists under the terms of the Creative Commons Attribution License (https://creativecommons.org/licenses/by/4.0/).

endolysosomal function, due to multiple effects on the pathways that modulate calcium concentration in the cuytosol.

Comments for the author

I am satisfied with the revisions and the new version of the manuscript.

Second revision

Author response to reviewers' comments

Reviewer 1 Comments for the Author:
The authors have responded to earlier comments, but incompletely.
Major original points:
1. The new data in Fig.1D-F helpfully indicate the normal scale of SOCE. OK

2. It seems incorrect to suggest that all reporters are acting within the 'quasi-linear' range. In the new panels (Fig 1D,E), for example, the response of GCaMP6S to CPA matches that produced by ionomycin (which very likely saturates the indicator). Furthermore, the F/F0 reaches about 35. Since the reported dynamic range of the indicator is ~60, and it is likely to be ~half-saturated at resting cytosolic [Ca2+] (F0), this too suggests saturation. Notwithstanding the limitations of calibrating fluorescence signals, I do not agree that the need to consider non-linear responses of indicators has been adequately addressed. The issue has clear implications. For example, the authors attach importance to the suggestion that the rate of change of pH and Ca2+ differ (p5), but that argument is not sustainable without taking account of the non-linear relationship between fluorescence and analyte.

We are sorry that the referee is not persuaded. However, we feel strongly that any potential reporter calibration (and/or saturation) do not affect our conclusions:

- If reporter saturation were an issue, we would not observe a prompt fall in the SOCE phase upon addition of the lysosomal agents (Figs. 3,4,5,6). Rather there would be a delay until Ca^{2+} fell within the reporters' 'linear' ranges. If anything, saturation would tend to *underestimate* the potency of the lysosomal agents (if there was a small, 'undetectable' Ca^{2+} fall). It would certainly not create an artefactual inhibition where there were none, so the effects we observe are real.
- In some experiments (e.g. Figs. 3,4,5), the SOCE phase is *lower* than the Ca^{2+}-release peak, meaning that SOCE cannot be saturated in these experiments.
- We see the same phenomenon using GEM-GECO1 (Fig. 6E-G) which has a *lower* affinity (K_d 340 nM) than GCaMP6s.
- Granted, calibration may somewhat alter the relative position of the intermediate concentration points in Figs. 4E-G, but this will be pretty marginal. We are careful never to claim that the *absolute* degree of inhibition of SOCE is equivalent to the inhibition of the GCaMP6s signal.
- We do not in fact attach 'much importance' to the discrepancy between the rate of change of pH and Ca^{2+}, we simply noted the discrepancy between the two:
  - We observed that the time-to-peak of a Ca^{2+} spike with nigericin is a few *seconds*, whereas the time-to-trough of pH is a few *minutes*.
  - Calibrating pH and Ca^{2+} will never change that discrepancy.
  - Regardless, it is a minor comment that we are happy to amend or remove.
- Bottom line: **we argue that calibration will neither dramatically improve nor alter the conclusion of the paper**.

3. OK

4. The legend to Fig. 5A remains unclear: although I figured it out, it should state absolutely clearly

© 2020. Published by The Company of Biologists under the terms of the Creative Commons Attribution License (https://creativecommons.org/licenses/by/4.0/).

which panels show rapolog and which FKBP proteins. I still do not see, from these panels, evidently all from thin sections that include most of the cell, where all the staining in control goes to after rapalog treatment. There is demonstrably vastly more staining in Ctrl then in either of the other panels. I don't think that either the revised text or Fig. S2 effectively addresses the issue. Why is there such a loss of LysoTracker staining after the rapalog treatments?

We are not sure what the referee means; rapalog is a *chemical* — none of the panels depicts this. FKBP is just one half of the chemical-induced dimerization system (the other half being FRB*). FKBP and FRB dimerize in the presence of rapalog.

If the referee means that they are confused as to which cells are treated with ethanol and which with rapalog, we now more clearly detail this in the Legend.

There is not a 'loss' of Lysotracker staining, there is a *redistribution and aggregation of lysosomes* (either at the MTOC or the periphery). Driving lysosomes to the MTOC in particular is *so* dramatic and extensive that the cytoplasm is, in effect, cleared of lysosomes that aggregate predominantly at 'one point'; the same thing is observed with the lysosomal marker-protein, LAMP1, in Fig. S2 which is *independent* of Lysotracker staining (hence we included this). This is explained in the text and referred to in the original 2015 paper.

Identical endo-lysosome redistribution patterns have been observed using optogenetic dimerization to motors (van Bergeijk et al., 2015), *Peri* (c) and *MTOC* (f) respectively:

[NOTE: We have removed unpublished data that had been provided for the referees in confidence.]

5. In quantifying responses of STIM1 in Fig. 6, the authors use STIM1 length. It is not clear to me what this means. Is this an inverse reporter of puncta formation? I acknowledge that the other measures provided in Fig. S3 provide alternative supportive quantification. The first 3 columns (basal and CPA) of Fig. 6A are all effectively replicates (ie 3 basal, and 3 store-depleted cells), yet the within-column variability is as great as that between the columns. In short, I find the images in Fig. 6A unconvincing (they are more convincing in Fig. 6D)
We apologise for not clarifying what length means. In resting cells, Stim1 morphology is ER/microtubular and in *long* connected structures. Puncta, by definition, are *shorter*, quasi-circular structures. We have simply measured the length (in µm, as detailed) before and after treatments. We have added the expression 'elongated structures' to reinforce that *basal* structures are longer.

We are sorry that the referee still finds the images unconvincing. We maintain that, for each single cell, the paired effects of CPA and the lysosome agents are clear.
However, we concede that the basal Stim1 morphology examples were somewhat variable, so we have now included an example of a different cell treated with LLOMe as a better match to the others. We hope that this is now acceptable.

6. OK on quantification. The observations in Fig 7 and 8 remain largely phenomenological, but that may be the nature of the beast at this stage.

van Bergeijk, P., Adrian, M., Hoogenraad, C. C. and Kapitein, L. C. (2015). Optogenetic control of organelle transport and positioning. *Nature* **518**, 111-4.

Third decision letter

MS ID#: JOCES/2020/248658

MS TITLE: Lysosomal agents inhibit store-operated Ca^{2+} entry

AUTHORS: Anthony Morgan and Antony Galione
ARTICLE TYPE: Research Article

© 2020. Published by The Company of Biologists under the terms of the Creative Commons Attribution License (https://creativecommons.org/licenses/by/4.0/).

I am happy to tell you that your manuscript has been accepted for publication in Journal of Cell Science, pending standard ethics checks.

© 2020. Published by The Company of Biologists under the terms of the Creative Commons Attribution License (https://creativecommons.org/licenses/by/4.0/).
